# Predicting potential global distribution and risk regions for potato cyst nematodes (*Globodera rostochiensis* and *Globodera pallida*)

**DOI:** 10.1038/s41598-022-26443-0

**Published:** 2022-12-17

**Authors:** Yitong He, Rui Wang, Honghai Zhao, Yonglin Ren, Manjree Agarwal, Dan Zheng, Shan Gao, Simon J. McKirdy, Dong Chu

**Affiliations:** 1 Shandong Engineering Research Centre for Environment-Friendly Agricultural Pest Management, College of Plant Health and Medicine, Qingdao Agricultural University, Qingdao, 266109 People’s Republic of China; 2 Harry Butler Institute, Murdoch University, Perth, WA 6150 Australia; 3 State Key Laboratory for Biology of Plant Diseases and Insect Pests, Institute of Plant Protection, Chinese Academy of Agricultural Sciences, Beijing, 100193 People’s Republic of China; 4 College of Economics, Qingdao Agricultural University, Qingdao, 266109 People’s Republic of China; 5 Hebei Dahaituo National Nature Reserve Management Centre, Chicheng, 075500 People’s Republic of China

**Keywords:** Biogeography, Invasive species, Ecological modelling

## Abstract

Potato cyst nematodes (PCNs), golden (yellow) cyst nematode (*Globodera rostochiensis*, gPCN) and pale (white) cyst nematode (*G. pallida*, pPCN), are important invasive pests in many countries and regions where they can cause significant yield and economic loss for agriculture. Prediction and identification of habitats suitable for PCNs are critical for developing biosecurity strategies, both pre and post border, to maximise the potential for early elimination should an incursion occur. To date, the potential global distribution of PCNs has not been thoroughly studied. Therefore, this study conducted a species distribution model to illustrate the potential global distribution of PCNs and risk regions. In this study, the Maximum Entropy Model (Maxent) associated with the Geographic Information System (GIS) was employed to reveal the potential distribution of the gPCN and pPCN. In addition to bioclimate, soil quality was also included in the model. The global cultivated lands, whether the susceptible hosts were present or not, were used to assess the maximum potential risk regions. The limitation factors for PCNs distribution were also assessed. Results showed that 66% of the global land surface was suitable for gPCN or pPCN or both, and both species can colonise more than 75% of the global cultivated lands. The coldest quarter’s mean temperature and precipitation were critical limitations in unsuitable regions. In summary, the global risk maps of PCNs contribute valuable additional information that complements previous national/regional distribution predictions. The results of this distribution research will contribute practical support for decision-makers and practitioners to implement biosecurity strategies from a global perspective, that incorporate prevention or promptly enforce control practices to limit the damage caused by future incursions.

## Introduction

Potato cyst nematodes (PCNs) are sedentary endoparasitic phytonematodes, including the golden (yellow) cyst nematode (*Globodera rostochiensis*, gPCN) and the pale (white) cyst nematode (*Globodera pallida*, pPCN). It is acknowledged that PCNs originated in South America and were introduced to Europe with potato breeding materials in the mid-nineteenth century^1,2^; subsequently, Europe became the secondary spread centre^3^. Currently, PCNs are present on virtually all continents (except Antarctica), while gPCN and pPCN have been reported from 74 and 49 countries or regions, respectively^4,5^. In Switzerland, a regular survey has been implemented annually since 1958, which has been continuously reporting the presence of PCNs^6^. Minnis et al. were the first to report a detailed field survey of PCNs in the United Kingdom, which identified severe infestation of both gPCN and pPCN^7^. In recent years, countries in most global regions have confirmed the presence of PCNs; these included Portugal, Turkey, Algeria, Morocco, India, Indonesia, Australia, Colombia, Kenya, Canada and USA^8–17^.

In the infested field, PCNs can cause significant damage to agricultural production. For example, in Europe, approximately nine per cent of potato yield reduction is a consequence of PCNs infection, with total crop failure occurring in some heavily infested areas^17^. Despite potatoes being one of the most economically important hosts, PCNs can parasitise more than 170 species in the *Solanaceae* family, including tomato and aubergine, which are also of high economic value^18^. Economic assessment in Australia has estimated that gPCN was predicted to cause an economic loss of AU$ 11.9 million to AU$ 27.0 million per year^19^, while in Idaho (USA), pPCN caused approximately US$26 million net loss in total agricultural output in 2006^20^. Moreover, the severe impact of PCNs is also reflected in the difficulties of management. The PCN disease epidemic often takes a long time (> 30 years) to be recognised after its first infestation and is hard to eradicate once it is detected in fields^21^. The environmental adaptability of PCNs makes them even more challenging to control, given their ability to survive without hosts for periods up to 30 years^4,22^, and their tolerance to a broad range of cold/warm temperatures^4,5,23^ (Supplementary Fig. S1).

Due to the widespread dispersal, severe damage, and the difficulties in management, a better understanding of PCNs potential distribution is important in order to facilitate biosecurity strategies, early detection and elimination. Some research focused on the regional or national spatial distribution patterns of PCNs have been published. Li predicted the potential distribution in China for both gPCN and pPCN with different habitat suitability models (HSMs)^24^, and Contina et al. used spatial algorithms to illustrate the pPCN dispersal pattern in Idaho (USA)^25^. However, few studies have elaborated on the potential global distribution of gPCN and pPCN. Thus, this study will focus on the PCNs distribution from a global perspective. The recognition of the global risk regions also provides a critical reference for the development of national/regional biosecurity strategies.

Model assessment is an efficient strategy to predict the potential distribution of a species^26^. Species distribution models (SDMs), also known as habitat suitability models (HSMs), are commonly used to reflect the species’ realised niche with survey data or historical records^26^. Maximum entropy (Maxent) is one of the advanced SDMs, based on machine learning and mathematical statistics^27^. The Maxent model possesses many advantages over its counterparts; for example, it does not require abundant ecological data of the targeted species and can use presence-only data to achieve high performance^28^. Maxent can also support multiple data formats of environmental variables, which can be continuous or categorical^29^. Given the uncertainty of absence records of PCNs and the various formats of environmental predictors, Maxent is an appropriate modelling method for this study.

In this study, the Maxent model associated with the Geographic Information System (GIS) was introduced to reveal the potential global distribution of gPCN and pPCN. Two assumptions were made to build up the foundation of this model. Firstly, this study assumed that either gPCN or pPCN would have an equal probability of presence and absence on the globe because both nematode species have adapted to almost all types of climates, including tropical, arid, temperate, continental, and tundra zones (Supplementary Fig. S1). The model, therefore, used the entire global land surface (except Antarctica) as background and predicted a maximum environmentally suitable range. This excluded the presence or absence of susceptible crop hosts. Secondly, this study assumed that all cultivated lands are potentially susceptive. Although potatoes, tomatoes or aubergines were not planted in all fields, they are possible to be planted on most of the cultivated lands due to their climate adaptation and cultivar diversity^30–32^. It is even true when taking facility agriculture into account, with which the hosts can be planted in cultivated lands even without suitable environments; therefore, PCNs can potentially distribute there. Moreover, the broad host range (> 170 species) of PCNs also included several regional vegetables and breeding materials, such as *Lycopersicon pimpinellifolium*, *Physalis longifolia*, and *Solanum aethiopicum*^4,5^, which increases the possibility of PCNs invasion in an expansive cultivated land. Thus, this study used the entire global cultivated land to predict the maximum potential risk regions. In conclusion, this paper aims to provide valuable information on the maximum potentially threatened region of PCNs invasion and a global reference for prevention and control strategies.

## Materials and methods

### Species presence data

The presence locations of gPCN and pPCN were gathered from web datasets, including Global Biodiversity Information Facility (http://www.gbif.org), Center for Agriculture and Bioscience International (http://www.cabi.org/isc) and European and Mediterranean Plant Protection Organization (http://www.eppo.int), as well as published government documents^33^. Primarily, 170 and 79 distribution points were collected for gPCN and pPCN, respectively. Those presence records without geographical coordinates were searched by names on Google Earth (https://earth.google.com/web/) to add longitude and latitude. However, sample points need to be selected considering some points may disperse intensively in the same raster cell, which can cause sample bias and strongly affect the model’s prediction^34^. Thus, the PCNs occurrence points were trimmed by a grid map of 10 × 10 km cells (5 arcminutes which is the same resolution as environmental variables) so that only one point is present per cell, ensuring that every point has distinct environment values^35^. Overall, 167 points for gPCN and 79 for pPCN were used for distribution prediction.

### Environmental variables

Suitable environmental conditions are the main factor for species occupation and the foundation of species distribution modelling (SDM)^26^. Bioclimate variables, such as temperature and precipitation are crucial and direct predictors for geographic distribution on large scales. Therefore, nineteen bioclimate variables with continuous data format were acquired from the WorldClim dataset (https://worldclim.org). Since PCNs are soil-dwelling species, soil quality variables were also introduced in this study to model the potential distribution of gPCN and pPCN. Thus, a soil quality variable with a categorical format was collected from the Harmonized World Soil Database (HWSD, https://www.fao.org/soils-portal/data-hub/soil-maps-and-databases/harmonized-world-soil-database-v12/en/). All environmental layers had the same resolution of five arcminutes.

However, not all environmental variables are necessary for prediction because some correlated attributes may contain similar information and lead to model overfitting. Thus, the Pearson coefficient was calculated to eliminate multicollinearity (r > |0.8|) for a better response between environmental variables and geographical distribution (Supplementary Fig. S2a). Then, two sets of environmental variables were used to select the optimal model (Table 1). Variables in the first set were selected by Jackknife analysis for their importance (Supplementary Fig. S2b,c). The second set included six environmental variables based on biological and ecological characteristics of PCNs^36^.

**Table 1:** Two sets of environmental variables for model selection.

		Label	Type	Description
First set		Bio8	Bioclimate	Average temperature of wettest quarter
	Second set	Bio10	Bioclimate	Average temperature of warmest quarter
		Bio11	Bioclimate	Average temperature of coldest quarter
		Bio12	Bioclimate	Annual precipitation
		Bio17	Bioclimate	Precipitation of driest quarter
		Bio19	Bioclimate	Precipitation of coldest quarter
		Sq1	Soil quality	Nutrient availability (integrated soil texture, soil organic carbon, soil pH, total exchangeable bases)

### Maxent modelling

Maxent (Version 3.4.1)^37^ was applied in this study to predict the environmentally suitable global distribution. Herein, different feature combinations and regularisation multipliers (β) were tested to optimise the model’s performance^38,39^. Maxent contains five features to illustrate the species-environment response (linear (l), quadratic (q), product (p), threshold (t) and hinge (h)). This study considered all possible feature combinations (31 in total). The regularisation multiplier varied between 0.5, 1, 2, and 3. In all, 248 candidate models have been evaluated for each species, with parameters reflecting all combinations of two sets of environmental variables, 31 feature class combinations, and four regularisation multiplier settings. Additionally, 80% of present localities were randomly selected for modal training, and the remaining 20% of localities were an independent test set to evaluate the model. The models were run with five replicates by cross-validation method, and the averaged results were automatically achieved via the programme.

The *kuenm* package^40^ in R (version 4.1.2)^41^ was applied to write a batch run script for model calibration and evaluation. The Akaike information criterion corrected for small sample sizes (AICc) and the partial area under the receiver operating characteristic curve (pROC) were used as performance metrics^42,43^. An omission rate of 5% was taken as the pROC calculation threshold. The candidate model with the lowest AICc value was taken as the best-performing final model. If there were candidate models with equal AICc values, the one with the highest pROC was selected as the final model.

### Result visualisation and spatial analysis of the risk area

ArcMap (version 10.4.1)^44^ was employed to visualise the prediction results. The results were reclassified to obtain environmentally suitable regions based on the Fixed Cumulative Value 5 (FCV5) because it is robust to abnormal values and prevalence, and balances the omission error at 5%^45^. The logistic predictions lower than FCV5 were defined as unsuitable, while those higher than FCV5 were suitable. Then, the appropriate area (logistic prediction over FCV5) for gPCN and pPCN was overlapped to define the suitable habitat for both species. Furthermore, the environmentally suitable regions were overlapped with cultivated land to generate the risk map. The global land-use map was collected from the FAO database^46^. The pixels with a cropland proportion above 5% were recognised as cultivated land. Then, the risk areas were classified into four levels (high risk, upper-medium risk, lower-medium risk, and low risk) based on the probability of observations^26^. Finally, all maps were projected to the World Geodetic System-1984 (WGS84) Coordinate System.

### Analyses of environmental response and limitations

First, the contribution and importance of each variable were analysed. The contribution of each variable was determined by the accumulated gain of each iteration in the training process. The variable importance was evaluated by calculating the area under the receiver operating characteristic curve (AUC)^47^. Then the variables that limited potato cyst nematodes distribution in unsuitable areas were assessed. The environmental variable’s response curves were used to determine each environmental variable’s better-preformed interval (Cloglog value > 0.5)^47^. The limitation factors were evaluated by the percentages of unsuitable areas dropped inside and outside the better-preformed interval.

## Results

### Model calibration and evaluation

In this study, 496 candidate models for gPCN and pPCN were calibrated (Supplementary Table S1 and Table S2). For gPCN candidate models, the AICc values varied from 4439.49 to 5287.05. However, two models equally obtained the lowest AICc value. The best-performed model (M_2_F_t_Set_1) was chosen by a higher pROC value (1.422), which used the multiplier *two*, feature *t*, and the first environmental variable set (Bio8, Bio10, Bio11, Bio12, Bio17, Bio19, and Sq1). The omission rate (OR) of gPCN’s best-performed model was 21%. For pPCN candidate models, the AICc values varied from 2078.58 to 14,063.16. The best-performed model (M_3_F_lqh_Set_1) used the multiplier *three*, feature combination *lqh*, and the first environmental variable set. This best-performed model’s pROC and OR were 1.5 and 26%, respectively. Although some candidate models got relatively low OR (lower than 5%), they also gained higher AICc and lower pROC than the selected final models, which indicated a low goodness-of-fit.

### Environmentally suitable habitats for potato cyst nematodes

**Figure 1:**
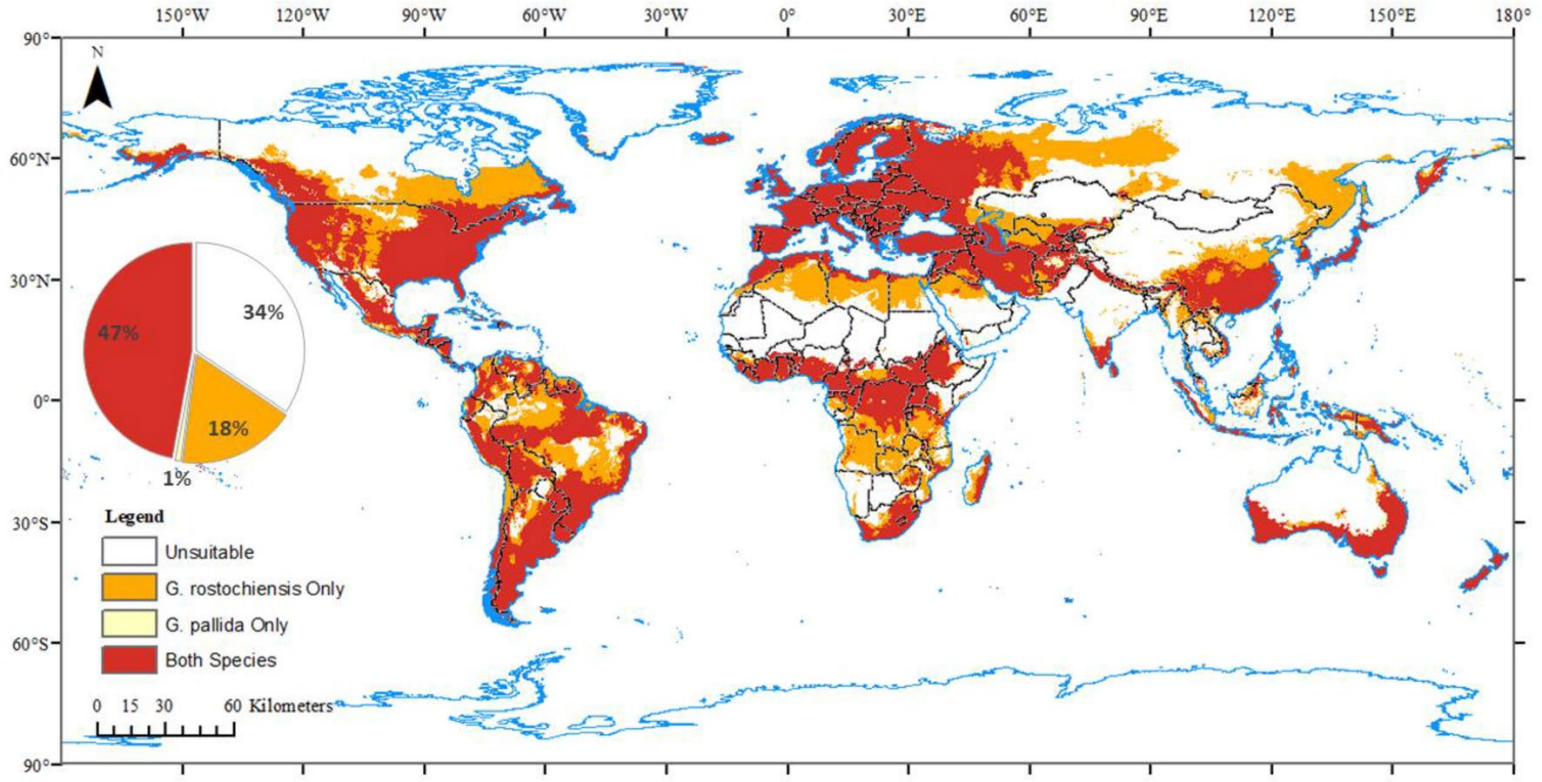
Environmentally suitable habitats for *Globodera rostochiensis* and *G. pallida* worldwide. White regions are unsuitable for both *G. rostochiensis* and *G. pallida*. Orange represents the areas only suitable for *G. rostochiensis*, while yellow regions are only suitable for *G. pallida*. Red areas are suitable for both *G. rostochiensis* and *G. pallida*. The pie chart shows the proportion of each suitable range on the entire global land surface. Global bioclimate data were acquired from the WorldClim open database (https://worldclim.org). The locations of species occurrence were collected from open databases: GBIF (http://www.gbif.org), CABI (http://www.cabi.org/isc) and EPPO (http://www.eppo.int). The species distribution model was conducted with Maxent (Version 3.4.1, http://biodiversityinformatics.amnh.org/open_source/maxent/), and results were modified with ArcMap (version 10.4.1, https://www.arcgis.com/).

The Maxent prediction indicated that the environmentally suitable habitats for both gPCN and pPCN are widely spread across the globe (Fig. 1). More than half (66%) of the land surface (excluding Antarctica) was suitable for at least one potato cyst nematode species. The areas suitable for both gPCN and pPCN were predicted to cover approximately 47% of the global land surface. The study identified that suitable habitats for both species could occupy the entire European continent, spreading from the British Isles to Iceland, extending to the Middle East and the Mediterranean coast of northern Africa. On the African continent, the suitable region also occupied the central and the southernmost points. The suitable area in North and South America expanded inland from the west and east coasts, taking approximately one-third of the continents. As for Asia, the suitable regions for both species were mainly distributed in southern China, the Korean Peninsula and Japan. Most suitable territories for the two nematode species in Oceania were distributed in the south and east coastal areas of Australia, and New Zealand.

The area only suitable for gPCN covered nearly 18% of the global land surface, which expanded along the boundaries of the area suitable for both species. These areas included central and eastern North America, central South America, the plateau of Eastern Europe and West Siberian, northern and central Africa, as well as central China and the region of Russia bordering northeast China. Moreover, the global land cover only suitable for pPCN was less than 1%, which indicated that over 97% of pPCN’s suitable habitats might be overlapped by gPCN’s habitats.

### Risk regions for potato cyst nematodes

**Figure 2:**
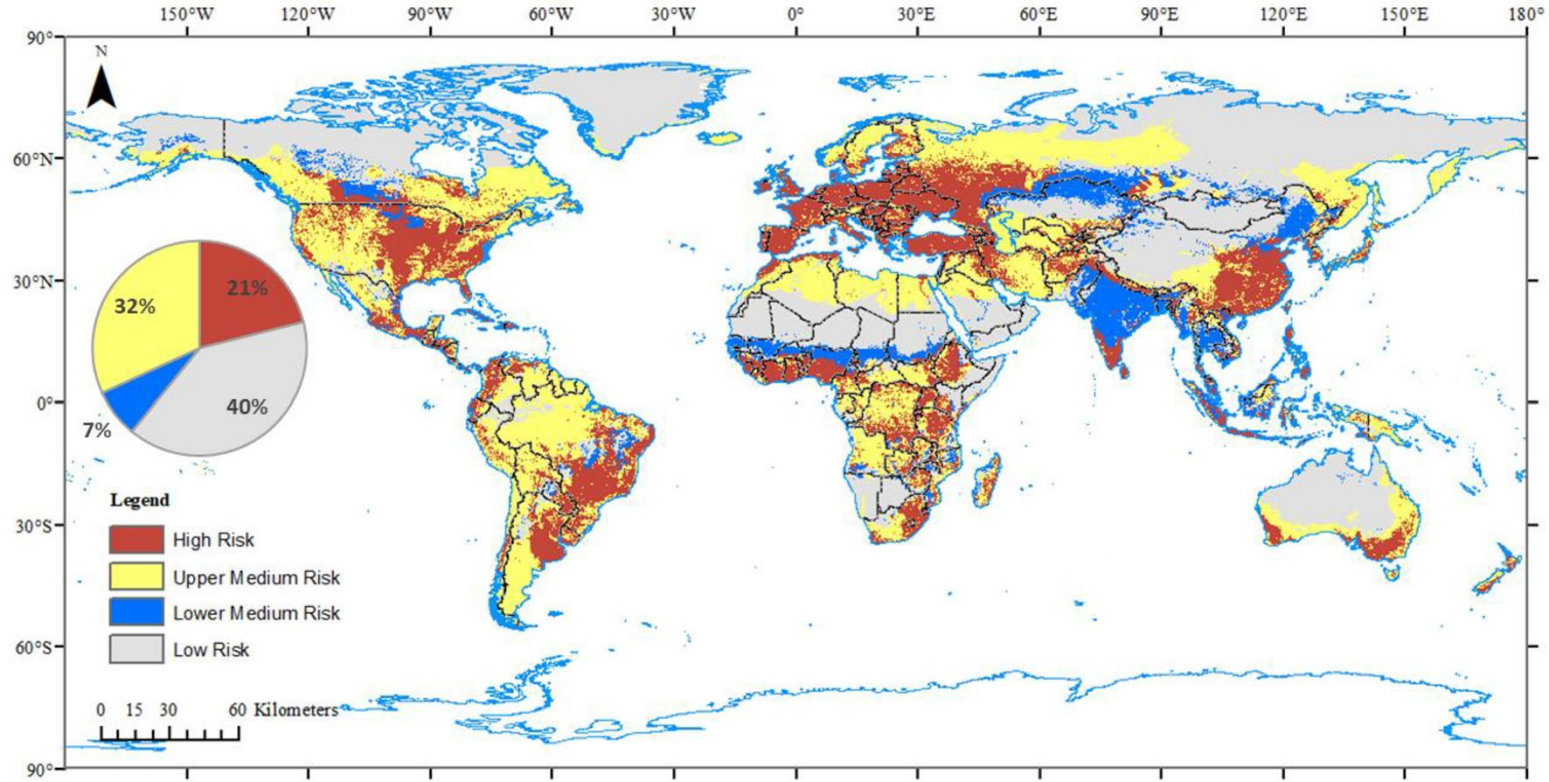
Global risk regions for *Globodera rostochiensis* and *G. pallida*. Grey areas have neither suitable environment nor cultivated land. Blue areas represent cultivated land with unsuitable environmental conditions. Yellow regions represent suitable environment with small areas of cultivated fields. Red areas are cultivated lands with suitable environments for at least one of *G. rostochiensis* and *G. pallida*. The pie chart shows the proportion of each risk level on the entire global land surface. Global bioclimate data were acquired from the WorldClim open database (https://worldclim.org). The locations of species occurrence were collected from open databases: GBIF (http://www.gbif.org), CABI (http://www.cabi.org/isc) and EPPO (http://www.eppo.int). The species distribution model was conducted with Maxent (Version 3.4.1, http://biodiversityinformatics.amnh.org/open_source/maxent/), and modified with ArcMap (version 10.4.1, https://www.arcgis.com/).

The risk map (Fig. 2) illustrated that the high-risk regions (the cultivated land with suitable environments for either gPCN or pPCN) covered 21% of the global land surface. These high-risk regions accounted for approximately 75% of global cultivated land. In contrast, the low-risk regions, which have neither suitable environment nor cultivated land, occupied 40% of the land cover.

The medium-risk areas were subdivided into two levels. Those environmentally suitable regions with only small areas of cultivated lands were defined as the upper-medium risk regions, while the cultivated land distributed in unsuitable environmental conditions was defined as lower-medium risk regions. Specifically, the upper-medium risk regions occupied 32% of the global land surface. Lower-medium risk regions occupied only 7% of the land surface.

### The environmental limitations of potato cyst nematodes distribution

The analyses of variables’ contribution and importance (Table 2) indicated that the mean temperature of the coldest quarter (Bio11) is the most critical variable in either gPCN or pPCN distribution prediction. The mean temperature of the wettest quarter (Bio8) took second place, while the mean temperature of the warmest quarter (Bio10) was the least important predictor among the three temperature variables. As for precipitation predictors, precipitation of the coldest quarter (Bio19) contributed the most in both models, especially in pPCN distribution predictions (51.2% contribution). Annual precipitation (Bio12) was also an essential predictor, especially in the gPCN model (13.3% contribution). Although the soil quality variable (Sq1) showed a low proportion of importance, it was still an indispensable predictor, which gave contributions even higher than the precipitation of the driest quarter (Bio17). Particularly in the pPCN model, Sq1 contributed 7.5% of the gains.

**Table 2:** The contribution and importance of each variable in *Globodera rostochiensis* and *G. pallida* models.

Variable	*G. rostochiensis*		*G. pallida*	
	Contribution (%)	Importance (%)	Contribution (%)	Importance (%)
Bio8	26.1	30.8	7.2	22.9
Bio10	5.8	4.2	0.5	1.5
Bio11	38.6	52.6	31.1	56.6
Bio12	13.3	7.1	2.1	4.4
Bio17	0.4	0.4	0.4	0.3
Bio19	15	4.5	51.2	13.9
Sq1	0.8	0.4	7.5	0.3

**Figure 3:**
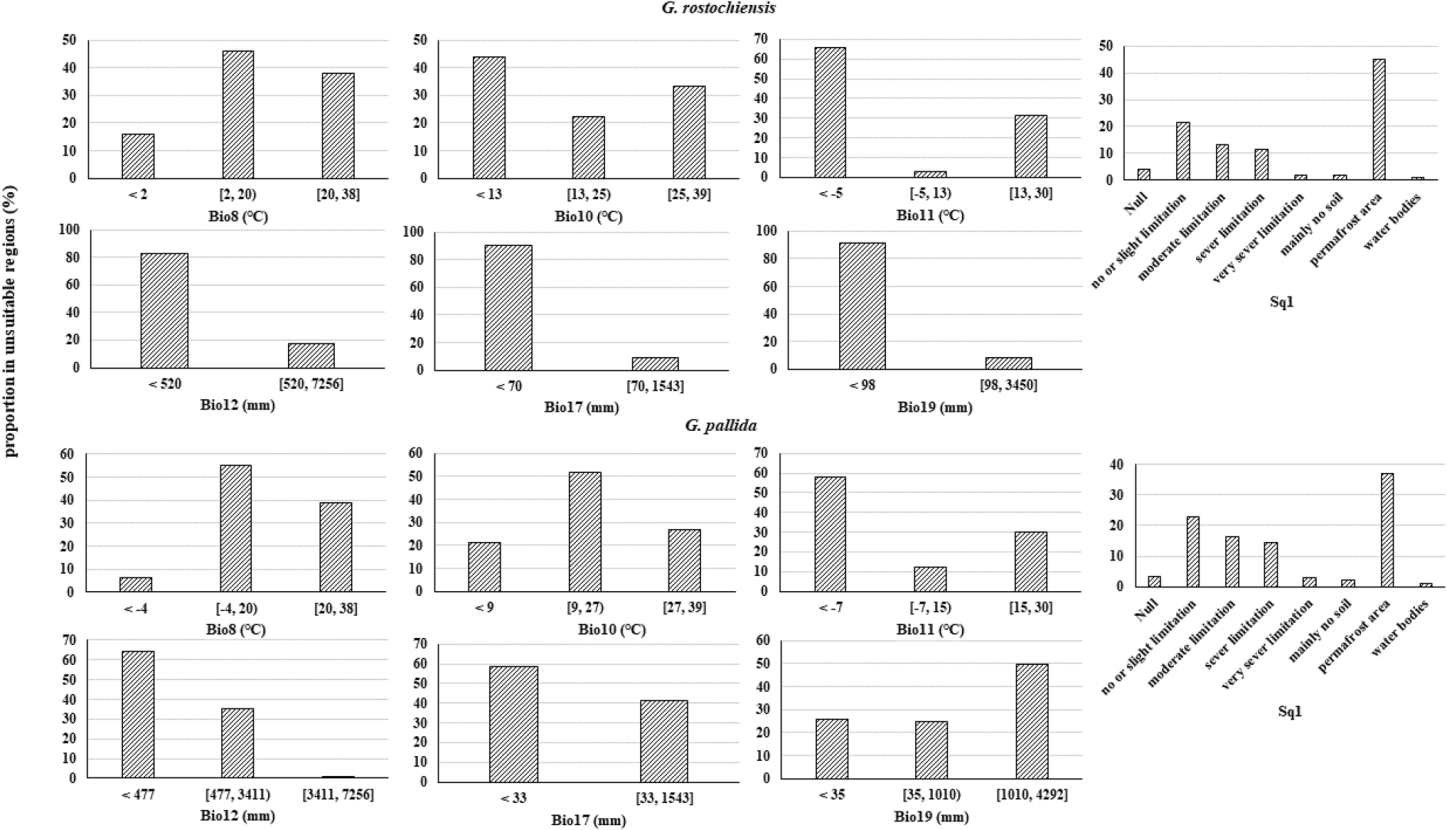
Histogram of environmental variables in unsuitable regions for both *Globodera rostochiensis* and *G. pallida*. *Bio8* average temperature of the wettest quarter, *Bio10* average temperature of the warmest quarter, *Bio11* average temperature of the coldest quarter, *Bio12* annual precipitation, *Bio17* precipitation of driest quarter, *Bio19* precipitation of coldest quarter, *Sq1* soil quality (integrated soil texture, soil organic carbon, soil pH, total exchangeable bases).

The analyses of limiting factors in unsuitable areas (Fig. 3) illustrated that the mean temperature of the wettest quarter (Bio8) did not suppress the distribution of gPCN and pPCN. 46% of the unsuitable regions still had a favourable temperature (2–20 °C) for gPCN at the wettest quarter. Similarly, 55% of pPCN’s unsuitable habitat had a favourable temperature (− 4 to 20 °C) at the wettest quarter. Except for Bio8, all the other environmental variables restricted the distribution of gPCN. In the warmest quarter, the temperatures of the most unsuitable habitats were either too low (< 13 °C) or too high (> 25 °C) for gPCN. In the coldest quarter, most unsuitable regions were too cold and dry, given that the temperature in 66% of the unsuitable land was lower than − 5 °C, and 92% of the land had precipitation less than 98 mm. Moreover, the annual precipitation also limited gPCNs distribution. Over 80% of the unsuitable regions had precipitation less than 570 mm, and 91% even less than 70 mm in the driest quarter. Unlike gPCN, the mean temperature of the warmest quarter did not restrict pPCN’s distribution. Over 50% of the unsuitable surface still had favourable temperatures (9–27 °C) for pPCN. The effects of other bioclimate variables on pPCN are similar to those on gPCN. It is worth noting that half of the unsuitable areas had too much precipitation (> 1010 mm) in the coldest quarter for pPCN. Soil quality affected the distribution of gPCN and pPCN in the same way. Most of the unsuitable regions were permafrost land; besides, around 30% of unsuitable land had limitations (from moderate to no soil) on soil quality.

## Discussion

Recent predictive modelling studies of species (plants, mammals, birds, insects) distribution have been based on climate variables or a combination of climate and topographic factors^26^. Unlike the insects above ground, nematodes complete their life cycle underground. Therefore, soil quality characteristics, such as soil texture and pH, may significantly affect nematode survival and spread^48,49^. Duyck et al. also acknowledged that soil type is an important variable in modelling nematode distribution^50^. Stanton et al. demonstrated that including land-use-related variables can achieve a more robust model prediction^51^. Soil quality is such a variable related to land characteristics and parameters. As such, this research utilised both bioclimate variables and soil quality factors along with cultivated land data to model the predicted distribution and risk regions of PCNs globally. As bioclimate information is continuous data, while soil quality is categorical data the Maxent model was selected as the preferred SDM due to its advantage in applying both data formats^52^.

Maxent’s tuning parameters are necessary to obtain higher confidence in species distribution prediction^28,38^. In this study, parameter combinations were identified in response to nonlinear species-environment curves with appropriate complexity^38^. The AICc is used as the selection criterion because it may ideally weigh the complexity of the estimated model against the goodness-of-fit^53^. Besides, the mean AUC ratio with partial ROC was used as a reference for model selection, which overcame the limitation that conventional AUC equally weighs omission and commission error^42^. As a result, the predicting model in this study provided satisfactory performance.

The selected model predicted that the environmentally suitable habitats for gPCN were relatively larger than pPCN’s and almost completely covered pPCN’s suitable territories, which indicted the niche similarity between these species. In reality, gPCN has been reported to occur together with pPCN in many areas. For example, 25% of the infested fields in England and Wales were detected with both nematode species, while the proportion was 28% in Algeria^7,8^. In Newfoundland (Canada), Portugal, and Kenya, gPCN was also accompanied by pPCN in some fields^11,14,16^.

A key feature of this research was that it focused on the entire global cultivated lands instead of just focusing on areas with known susceptible hosts, in order to provide results that predict the maximum potential of PCNs global distribution. The prediction considered the global plantation structure as a changeable rather than fixed system because the major hosts of PCNs (potato, tomato, and aubergine) have demonstrated that they can be planted in most cultivated lands, especially with anthropogenic management activities (e.g., breeding temperature and drought resistance cultivars, or building agricultural facilities such as greenhouse to improve planting conditions)^30–32^. Moreover, the global challenges in recent years (such as climate change, the COVID-19 pandemic, and the Russia-Ukraine war) have been influencing the food supply and planting patterns globally^54,55^.

This research provides a valuable contribution to future biosecurity management by predicting the distribution risk across cultivated and non-cultivated areas. The spread of gPCN and pPCN would be highly risky because PCNs’ environmentally suitable habitats covered most global cultivated lands, which meet the essential requirement of species colonisation and survival. Given that potatoes, the primary host of PCNs, are planted intensively in Europe, China, the southern Himalayas and the Andes region^56^, and many other regions of the globe, the invasion and spread of PCNs in these regions will initiate control/management strategies to address the risk. Furthermore, aubergine and tomatoes are also economically significant hosts^18^, which results in high risk in more extensive horticultural areas.

Global areas with a suitable environment but limited cultivated lands were considered upper-medium risk regions. Although high economic value hosts may not be present in these regions, PCNs could still colonise many *Solanaceae* plants^18^, which are present on all continents (except Antarctica)^57^. Cultivated lands without suitable environments should also be treated with caution because colonisation may happen via high propagule pressure^26^. Moreover, some hosts, such as tomatoes and aubergines, can be planted in greenhouses or other artificial conditions. Thus, even if the natural environment is unsuitable, these cultivated lands should be treated cautiously to prevent possible incursions. Also, anthropogenic effects (e.g., thriving trade of breeding materials) may exacerbate the risk of PCNs’ dispersal, so strict biosecurity strategies should be executed particularly at the boundary of each risk region.

The results of the unsuitable regions are not definitive because our model used the presence-only data, while biotic factors, such as interspecific competition, are difficult to access^38^. Therefore, our prediction of the suitable habitats is a comparable valuation of the ecological niche, and PCNs would establish in low suitability regions if they were to invade with large numbers and reproduce a substantial clutch size^58^. Moreover, human cultivation activities that modify land areas probably increase this possibility and could cause significant uncertainties in the low risk regions^59^. Despite these limitations in modelling the potential distribution, the results we present can provide fundamental and valuable information about different global regions with a higher possibility for PCNs to invade and establish. A greater understanding of the potential habitats achieved from these results can also help to assess the species’ bilateral flow and invasion pathways.

In this study, the environmental limitations showed that pPCN had a broader temperature adaptation than gPCN. The predicted suitable temperature range (from coldest to warmest quarter) for pPCN is − 7 to 27 °C, while the suitable temperature range for gPCN is − 5 to 25 °C. Jones et al. reported that the development and reproduction of female pPCN preferred 15–17.5 °C while gPCN favoured 17.5–22.5 °C^60^, which supported the temperature range predicted in this study. The results from Kaczmarek et al. showed a broader temperature range with a similar trend (i.e., 13–25 °C for pPCN and 15–27 °C for gPCN), and they also presented that when the temperature was over 25 °C pPCN hatched more efficiently than gPCN^61^. Noteworthy is that pPCNs may outcompete gPCNs in the mixed-species soil due to their faster and more significant multiplication^62^. The broader suitable temperature range for pPCN utilised in this study can also support its greater competitiveness than gPCN. In reality, pPCN has shown a trend of gradually replacing gPCN in Britain^21^. Thus, the area only suitable for gPCN should also be treated discreetly with pPCN invasion.

The environmental limitations analysis also showed that the coldest quarter’s mean temperature is the significant temperature-related bioclimate factor. However, with climate change, areas affected by this frigid climate may gradually shrink so that the suitable habitats for PCNs can be assumed to extend toward the poles and higher elevations. Some studies have pointed out that the increasing temperature can affect PCNs’ survival^60,63^, whereas whether the effect will be significant enough to shift their distribution still needs to be thoroughly studied. This study also indicated that precipitation significantly suppressed PCNs’ distribution in unsuitable regions. An arid climate is also likely to adversely affect PCNs distribution. However, studies have focused on the effect of temperature increases rather than precipitation changes^60,63^. Thus, the effect of precipitation on PCN’s ecological adaption and geographical distribution needs more thorough research, especially under the challenge of climate change.

Scientific precautionary management needs to be implemented to minimise the ecological, economic and social impact caused by pest invasion, in which the awareness of possible high-risk regions is a crucial element in biosecurity strategies^64^. Incorporating climate suitability and host availability, as well as other specific suitability criteria such as soil characteristics, are the primary steps, from which policy decision-makers can focus on pathway analysis and detection regimes at borders^65^. Since species invasion can significantly threaten global ecological systems and economies, knowledge of potentially suitable habitats is imperative to manage risk^66^. It is well accepted that elimination and control practices need to be implemented as early as possible in order to achieve desired biosecurity outcomes^67^, and predictive knowledge of potential distribution is a key element in this decision-making. This is particularly the case for nematode prevention as they are often overlooked due to symptoms on host plants not being readily obvious at the early stages of infestation^68^, and once established and detected any management response will consume enormous resources and the probability of elimination decreases. For these reasons, the prediction of risk regions will provide significant support to decision-makers assisting in the accurate and early detection of PCNs to overcome this challenge. This highlights the valuable contribution the potential global risk maps for PCNs presented in this study can provide for future biosecurity strategies and early elimination practices.

In conclusion, this study presented the potential globally suitable distribution of PCNs based on both climate and soil factors. The risk regions were predicted based on suitable environments along with global cultivated land and achieved a maximum potential range from the perspective of potential variation. The prediction is an informative complement for regional/national studies, which can support further biosecurity decision-making and strategies on PCNs. Additionally, soil quality played an indispensable role in PCNs distribution models and highlighted the need to include additional parameters relevant to the ecology of the species under consideration. The mean temperature in the coldest quarter and precipitation are the main environmental limitations for PCNs’ distribution, and the authors identified that the response of these nematodes to low temperature and soil moisture needs further study to further progress potential distribution modelling.

## Supplementary Information

Supplementary Information.

## Data Availability

All data generated or analysed during this study are included in this published article and its supplementary information files.
