# Supplementary material for: Predicting potential global distribution and risk regions for potato cyst nematodes (Globodera rostochiensis and Globodera pallida)
Source: Sci Rep. 2022 Dec 17;12:21843. doi: 10.1038/s41598-022-26443-0 (PMC9759053; doi:10.1038/s41598-022-26443-0)
Supplement: Supplementary file 1 — Supplementary Information. [file 41598_2022_26443_MOESM1_ESM.docx]

Predicting Potential Global Distribution and Risk Regions for Potato Cyst Nematodes (*Globodera rostochiensis* and *Globodera pallida*)

Yitong He^1, 2, *^, Rui Wang^3^, Honghai Zhao^1^, Yonglin Ren^2^, Manjree Agarwal^2^, Dan Zheng^4^, Shan Gao^5^, Simon J. McKirdy^2, *^ and Dong Chu^1, *^

^1^ Shandong Engineering Research Centre for Environment-friendly Agricultural Pest Management, College of Plant Health and Medicine, Qingdao Agricultural University, Qingdao 266109, P. R. China; [hhzhao@qau.edu.cn](mailto:hhzhao@qau.edu.cn) (H. Z.)

^2^ Harry Butler Institute, Murdoch University, Perth, WA 6150, Australia; y.ren@murdoch.edu.au (Y. R.); M.Agarwal@murdoch.edu.au (M. A.)

^3^ State Key Laboratory for Biology of Plant Diseases and Insect Pests, Institute of Plant Protection, Chinese Academy of Agricultural Sciences, Beijing, 100193, P. R. China; wangrcaas@163.com

^4^ College of Economics, Qingdao Agricultural University, Qingdao 266109, P. R. China, zhengdan1818@163.com

^5^ Hebei Dahaituo National Nature Reserve Management Centre, Chicheng 075500, P. R. China; jialugao@foxmail.com

* Corresponding authors: chinachudong@qau.edu.cn (D. C.); s.mckirdy@murdoch.edu.au (S. M.); hoyt.he@murdoch.edu.au (Y. H.)

**Supplementary Information**


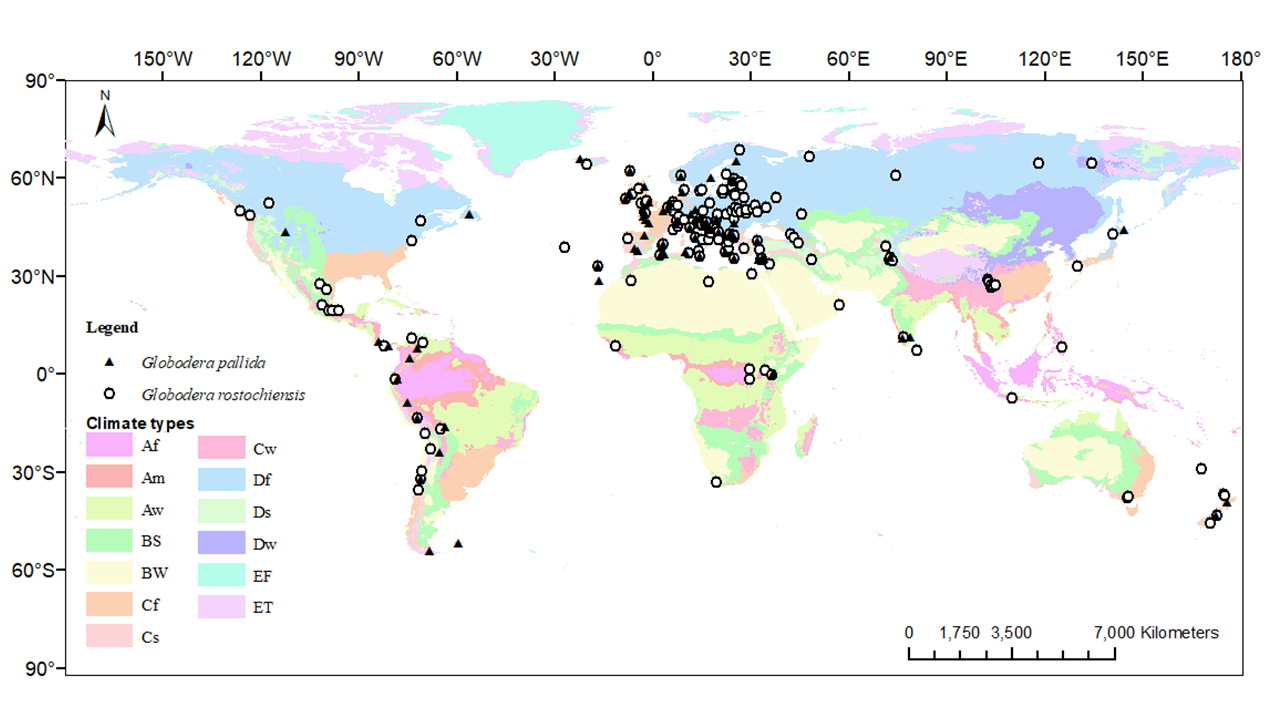


**Figure S1. The current distribution of *G. rostochiensis* and *G. pallida* in different climate types**. Colours represent different Koppen climate zones: Af, tropical without dry season; Am, tropical with a short dry season; Aw, tropical with a dry winter; BW, arid desert; BS, arid steeps; Cf, temperate regions wet all year; Cs, temperate regions with a dry summer; Cw, temperate regions with a dry winter; D, continental regions (f, s, w are the same as C); EF, snow and ice region; ET, tundra region. Koppen climate data were accessed from Climod (https://www.climond.org/Resources.aspx). The locations of species occurrence were collected from open databases: GBIF (http://www.gbif.org), CABI (http://www.cabi.org/isc) and EPPO (http://www.eppo.int). Species occurrence locations were collected from open databases: GBIF (http://www.gbif.org), CABI (http://www.cabi.org/isc) and EPPO (http://www.eppo.int). The map was generated with ArcMap (version 10.4.1, https://www.arcgis.com/).


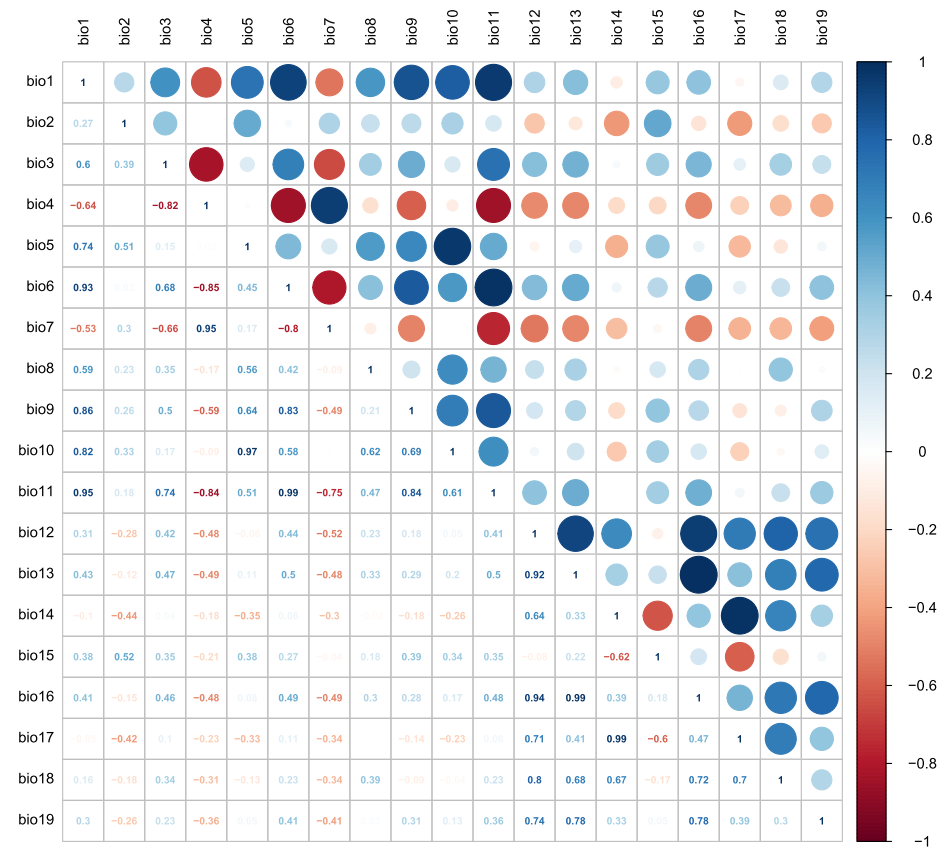


(a)


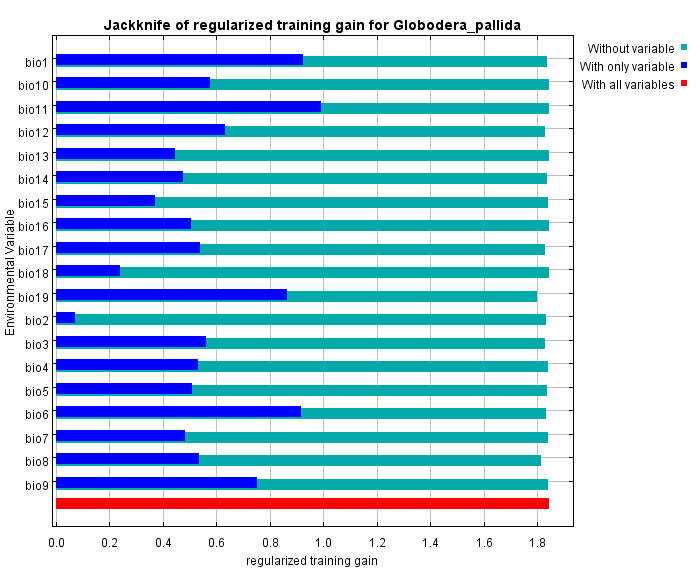


(b)


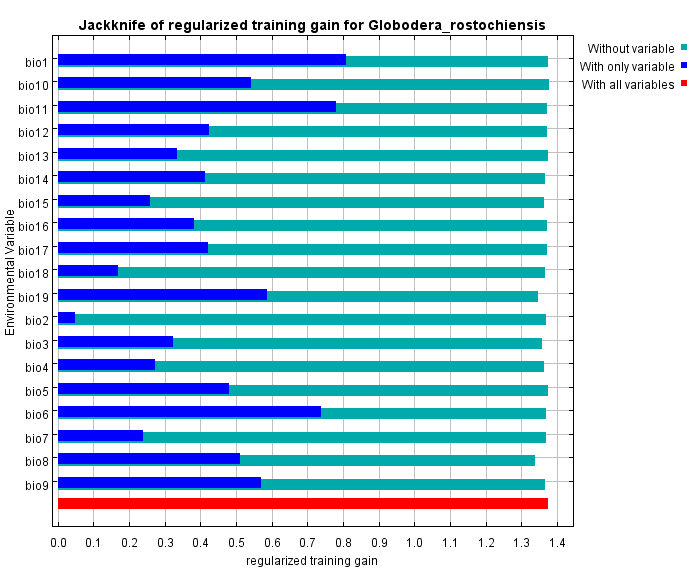


(c)

**Figure S2. Preliminary test to select bioclimate variables.** (a) Pearson correlations between 19 bioclimate variables. (b) Jackknife of regularized training gain for *Globodera pallida*. (c) Jackknife of regularized training gain for *Globodera rostochiensis*. Remove variables that Pearson coefficient r>|0.8|, referring to the importance in the jackknife test.


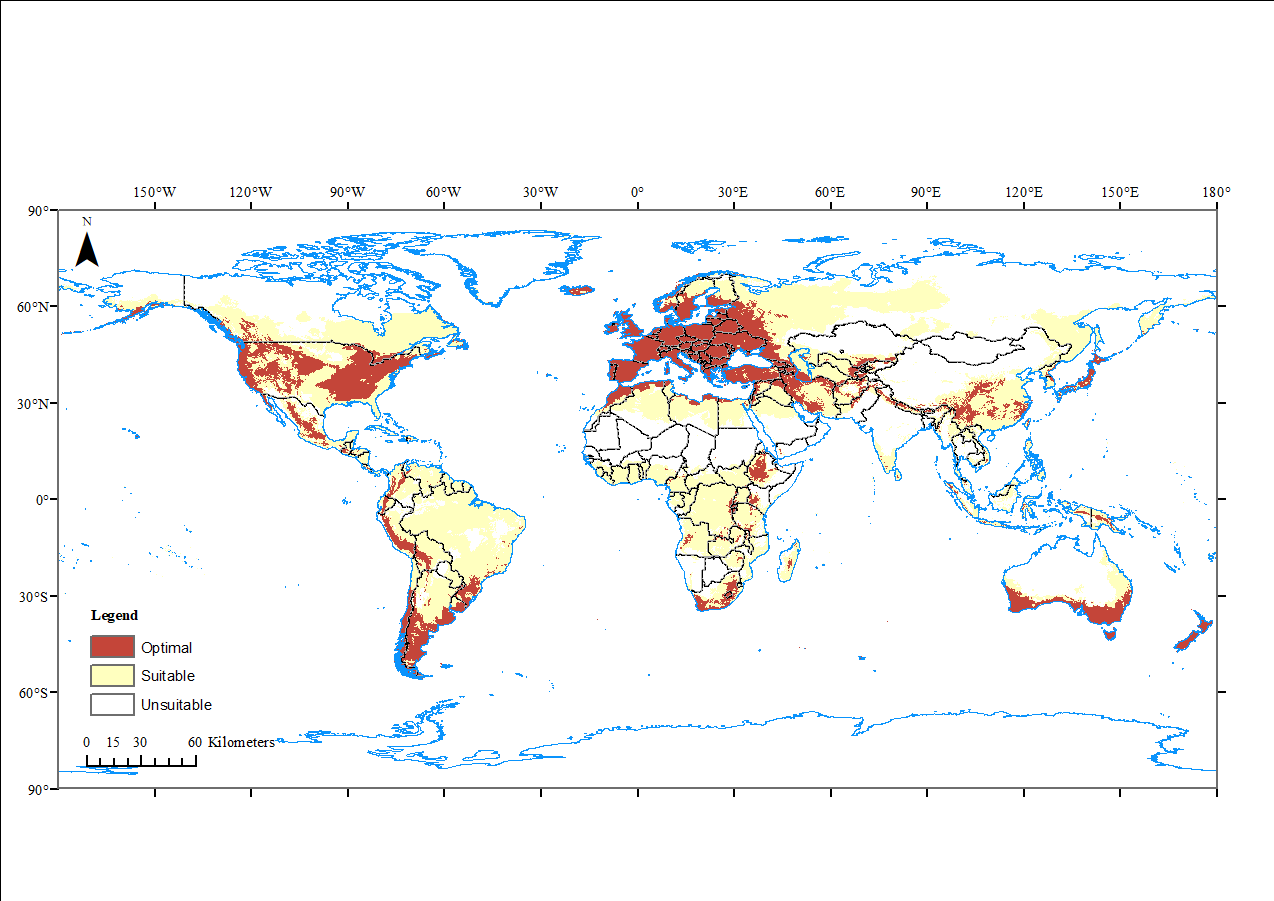


**Figure S3. Potential distribution of *G. rostochiensis* worldwide**. White represents unsuitable habitats, while yellow and red represent suitable and optimal habitats, respectively. The areas with probability lower than study Fixed Cumulative Value 5 (FCV5) were defined as unsuitable, while those higher than FCV5 were defined as suitable. The areas that obtained probability greater than Maximum Sensitivity Plus Specificity of Training Data (MSS) were defined as optimal. Global bioclimate data were acquired from the WorldClim open database (https://worldclim.org). The locations of species occurrence were collected from open databases: GBIF (http://www.gbif.org), CABI (http://www.cabi.org/isc) and EPPO (http://www.eppo.int). The species distribution model was conducted with Maxent (Version 3.4.1, http://biodiversityinformatics.amnh.org/open_source/maxent/) and modified with ArcMap (version 10.4.1, https://www.arcgis.com/).


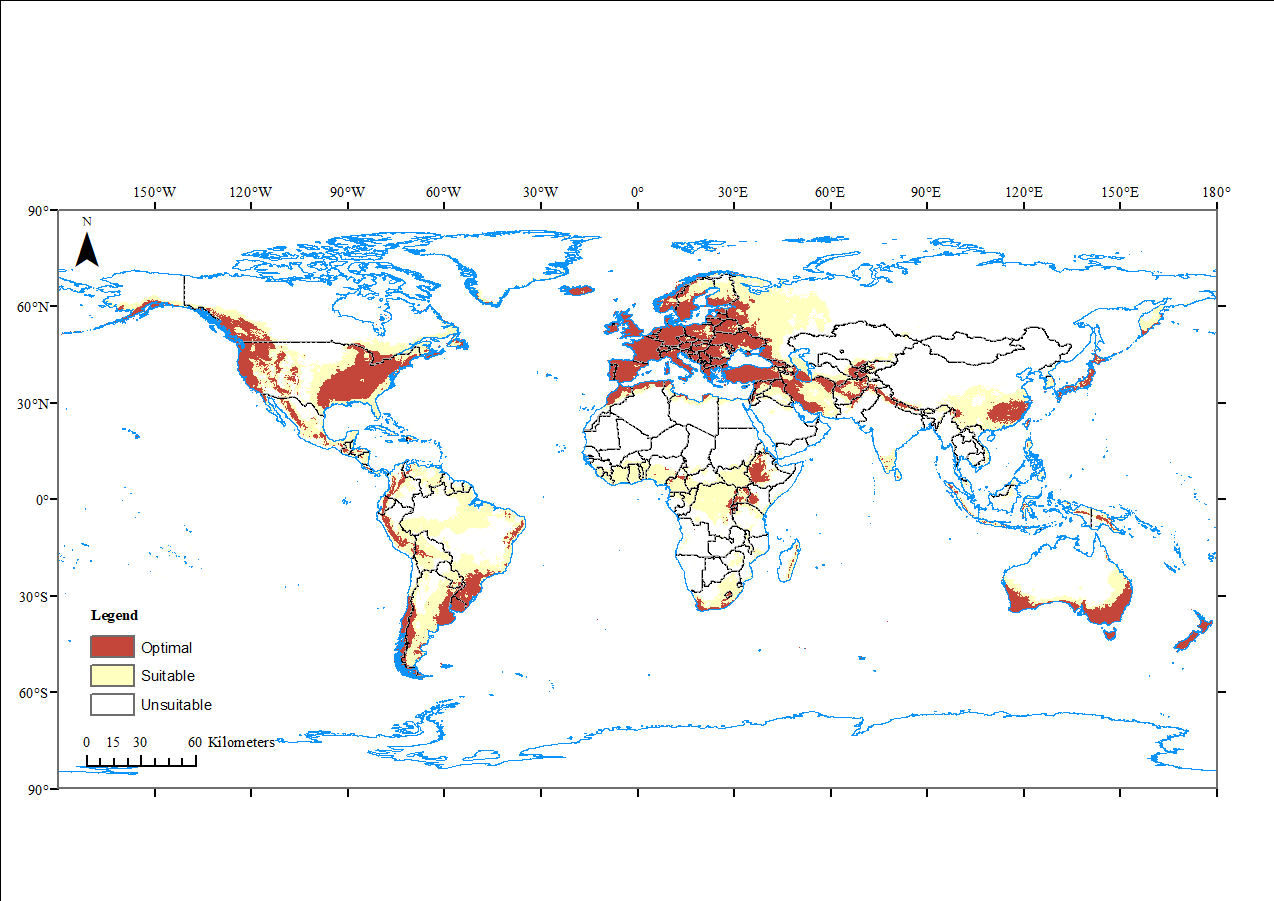


**Figure S4. Potential distribution of** ***G. pallida* worldwide.** White represents unsuitable habitats, while yellow and red represent suitable and optimal habitats, respectively. The areas with probability lower than study Fixed Cumulative Value 5 (FCV5) were defined as unsuitable, while those higher than FCV5 were defined as suitable. The areas that obtained probability greater than Maximum Sensitivity Plus Specificity of Training Data (MSS) were defined as optimal. Global bioclimate data were acquired from the WorldClim open database (https://worldclim.org). The locations of species occurrence were collected from open databases: GBIF (http://www.gbif.org), CABI (http://www.cabi.org/isc) and EPPO (http://www.eppo.int). The species distribution model was conducted with Maxent (Version 3.4.1, http://biodiversityinformatics.amnh.org/open_source/maxent/) and modified with ArcMap (version 10.4.1, https://www.arcgis.com/).

**Table S1 Maxent calibration results for *Globodera pallida*.** Totally 248 candidate models were evaluated. Candidate models were ranked by AICc. NA means the number of parameters was over the number of presence recorders.

| Model | Mean AUC ratio of pROC | Omission rate at 5% | AICc | Delta AICc | Number of parameters |
| --- | --- | --- | --- | --- | --- |
| M_3_F_lqh_Set_1 | 1.504 | 0.267 | 2078.580 | 0.000 | 17 |
| M_3_F_lqth_Set_1 | 1.537 | 0.267 | 2079.242 | 0.663 | 18 |
| M_2_F_lqh_Set_1 | 1.499 | 0.267 | 2080.592 | 2.013 | 22 |
| M_3_F_lqt_Set_2 | 1.501 | 0.200 | 2081.220 | 2.640 | 14 |
| M_3_F_lqh_Set_2 | 1.551 | 0.267 | 2081.596 | 3.017 | 16 |
| M_2_F_qt_Set_2 | 1.489 | 0.267 | 2082.357 | 3.777 | 17 |
| M_2_F_lqph_Set_2 | 1.537 | 0.267 | 2082.481 | 3.902 | 20 |
| M_2_F_pt_Set_1 | 1.458 | 0.267 | 2083.019 | 4.439 | 22 |
| M_2_F_t_Set_1 | 1.483 | 0.267 | 2085.331 | 6.751 | 24 |
| M_2_F_lqh_Set_2 | 1.533 | 0.267 | 2086.377 | 7.798 | 21 |
| M_2_F_lqt_Set_2 | 1.519 | 0.267 | 2086.600 | 8.020 | 20 |
| M_3_F_lqpt_Set_1 | 1.520 | 0.333 | 2087.047 | 8.467 | 17 |
| M_3_F_lt_Set_1 | 1.492 | 0.267 | 2087.272 | 8.692 | 19 |
| M_3_F_lqpt_Set_2 | 1.517 | 0.200 | 2087.774 | 9.195 | 16 |
| M_3_F_qt_Set_1 | 1.457 | 0.267 | 2088.200 | 9.621 | 16 |
| M_3_F_lqph_Set_1 | 1.506 | 0.267 | 2089.290 | 10.710 | 20 |
| M_3_F_qpt_Set_1 | 1.495 | 0.267 | 2090.097 | 11.518 | 18 |
| M_3_F_lqt_Set_1 | 1.506 | 0.333 | 2090.446 | 11.866 | 18 |
| M_3_F_lph_Set_1 | 1.471 | 0.267 | 2090.806 | 12.226 | 19 |
| M_3_F_t_Set_1 | 1.451 | 0.267 | 2090.900 | 12.321 | 16 |
| M_3_F_qth_Set_2 | 1.524 | 0.200 | 2091.020 | 12.440 | 19 |
| M_3_F_qpt_Set_2 | 1.523 | 0.267 | 2091.397 | 12.817 | 16 |
| M_3_F_lqpth_Set_1 | 1.490 | 0.267 | 2091.801 | 13.221 | 22 |
| M_3_F_qt_Set_2 | 1.477 | 0.267 | 2092.523 | 13.944 | 13 |
| M_3_F_ph_Set_1 | 1.495 | 0.200 | 2092.896 | 14.317 | 19 |
| M_2_F_lt_Set_1 | 1.484 | 0.267 | 2093.336 | 14.756 | 27 |
| M_2_F_lqph_Set_1 | 1.508 | 0.267 | 2093.415 | 14.835 | 25 |
| M_3_F_lth_Set_1 | 1.465 | 0.267 | 2093.931 | 15.351 | 23 |
| M_3_F_lqph_Set_2 | 1.539 | 0.267 | 2094.392 | 15.813 | 20 |
| M_2_F_lqpt_Set_2 | 1.530 | 0.267 | 2094.789 | 16.209 | 23 |
| M_3_F_lqth_Set_2 | 1.535 | 0.267 | 2096.054 | 17.474 | 21 |
| M_2_F_qt_Set_1 | 1.459 | 0.267 | 2096.405 | 17.825 | 24 |
| M_3_F_lqpth_Set_2 | 1.535 | 0.267 | 2097.957 | 19.378 | 22 |
| M_2_F_lpt_Set_2 | 1.486 | 0.267 | 2098.558 | 19.978 | 23 |
| M_3_F_lpt_Set_2 | 1.518 | 0.200 | 2098.972 | 20.392 | 17 |
| M_3_F_pt_Set_1 | 1.448 | 0.267 | 2099.329 | 20.750 | 19 |
| M_2_F_lpt_Set_1 | 1.469 | 0.267 | 2099.518 | 20.938 | 27 |
| M_3_F_lh_Set_1 | 1.468 | 0.267 | 2100.292 | 21.712 | 22 |
| M_0.5_F_lq_Set_1 | 1.445 | 0.267 | 2100.304 | 21.725 | 15 |
| M_2_F_lqpth_Set_2 | 1.544 | 0.267 | 2100.375 | 21.795 | 26 |
| M_3_F_h_Set_1 | 1.467 | 0.200 | 2100.642 | 22.063 | 21 |
| M_2_F_qpt_Set_2 | 1.555 | 0.267 | 2100.663 | 22.084 | 24 |
| M_0.5_F_lqp_Set_1 | 1.425 | 0.333 | 2100.718 | 22.138 | 22 |
| M_2_F_lt_Set_2 | 1.507 | 0.333 | 2100.812 | 22.232 | 25 |
| M_2_F_qpt_Set_1 | 1.492 | 0.267 | 2101.379 | 22.799 | 27 |
| M_1_F_lq_Set_1 | 1.433 | 0.267 | 2101.869 | 23.289 | 14 |
| M_1_F_lqp_Set_1 | 1.482 | 0.267 | 2102.894 | 24.315 | 18 |
| M_0.5_F_lqp_Set_2 | 1.422 | 0.267 | 2103.178 | 24.599 | 18 |
| M_3_F_lpt_Set_1 | 1.464 | 0.267 | 2103.199 | 24.619 | 21 |
| M_2_F_lq_Set_1 | 1.454 | 0.267 | 2105.190 | 26.610 | 12 |
| M_2_F_lqt_Set_1 | 1.508 | 0.333 | 2105.560 | 26.980 | 27 |
| M_3_F_lpth_Set_1 | 1.476 | 0.267 | 2106.078 | 27.498 | 25 |
| M_2_F_lqth_Set_2 | 1.541 | 0.267 | 2106.246 | 27.667 | 27 |
| M_3_F_lt_Set_2 | 1.518 | 0.267 | 2106.552 | 27.972 | 20 |
| M_2_F_lqpt_Set_1 | 1.516 | 0.333 | 2107.149 | 28.569 | 28 |
| M_1_F_lqp_Set_2 | 1.483 | 0.200 | 2107.746 | 29.166 | 15 |
| M_0.5_F_lq_Set_2 | 1.498 | 0.267 | 2108.055 | 29.475 | 12 |
| M_2_F_lph_Set_1 | 1.471 | 0.267 | 2108.210 | 29.630 | 28 |
| M_2_F_lqp_Set_1 | 1.493 | 0.267 | 2109.591 | 31.011 | 15 |
| M_3_F_lq_Set_1 | 1.462 | 0.267 | 2109.596 | 31.016 | 10 |
| M_3_F_qpth_Set_2 | 1.529 | 0.267 | 2110.150 | 31.571 | 24 |
| M_2_F_lqp_Set_2 | 1.494 | 0.200 | 2110.880 | 32.300 | 11 |
| M_0.5_F_qp_Set_1 | 1.475 | 0.267 | 2110.911 | 32.331 | 20 |
| M_3_F_pth_Set_1 | 1.465 | 0.267 | 2111.160 | 32.581 | 26 |
| M_2_F_t_Set_2 | 1.510 | 0.333 | 2111.356 | 32.776 | 26 |
| M_3_F_lqp_Set_1 | 1.460 | 0.267 | 2111.537 | 32.958 | 11 |
| M_1_F_qp_Set_1 | 1.496 | 0.200 | 2111.605 | 33.025 | 16 |
| M_2_F_lqth_Set_1 | 1.514 | 0.267 | 2112.030 | 33.450 | 30 |
| M_3_F_t_Set_2 | 1.500 | 0.333 | 2112.424 | 33.844 | 18 |
| M_1_F_t_Set_1 | 1.483 | 0.267 | 2112.674 | 34.094 | 38 |
| M_3_F_h_Set_2 | 1.501 | 0.267 | 2112.923 | 34.343 | 23 |
| M_0.5_F_qp_Set_2 | 1.442 | 0.267 | 2113.805 | 35.226 | 16 |
| M_2_F_pt_Set_2 | 1.500 | 0.200 | 2114.622 | 36.042 | 26 |
| M_2_F_qp_Set_1 | 1.489 | 0.200 | 2114.862 | 36.282 | 13 |
| M_3_F_lq_Set_2 | 1.477 | 0.267 | 2114.888 | 36.308 | 8 |
| M_3_F_lqp_Set_2 | 1.481 | 0.267 | 2115.094 | 36.514 | 9 |
| M_2_F_lq_Set_2 | 1.476 | 0.267 | 2116.142 | 37.562 | 11 |
| M_3_F_ph_Set_2 | 1.518 | 0.267 | 2116.545 | 37.965 | 24 |
| M_0.5_F_lp_Set_1 | 1.436 | 0.267 | 2116.641 | 38.062 | 19 |
| M_2_F_qp_Set_2 | 1.499 | 0.200 | 2116.795 | 38.215 | 10 |
| M_1_F_qp_Set_2 | 1.512 | 0.200 | 2116.803 | 38.224 | 14 |
| M_3_F_lh_Set_2 | 1.486 | 0.267 | 2116.923 | 38.343 | 24 |
| M_1_F_lq_Set_2 | 1.421 | 0.267 | 2117.610 | 39.031 | 14 |
| M_2_F_ph_Set_1 | 1.483 | 0.267 | 2117.760 | 39.180 | 29 |
| M_1_F_t_Set_2 | 1.523 | 0.333 | 2118.545 | 39.965 | 36 |
| M_2_F_h_Set_1 | 1.472 | 0.267 | 2119.363 | 40.783 | 29 |
| M_2_F_qth_Set_2 | 1.518 | 0.267 | 2120.238 | 41.658 | 30 |
| M_2_F_qpth_Set_2 | 1.514 | 0.267 | 2120.720 | 42.140 | 30 |
| M_3_F_pt_Set_2 | 1.513 | 0.200 | 2121.349 | 42.769 | 20 |
| M_1_F_lp_Set_1 | 1.432 | 0.267 | 2121.366 | 42.786 | 18 |
| M_3_F_th_Set_1 | 1.470 | 0.267 | 2121.378 | 42.798 | 28 |
| M_3_F_qth_Set_1 | 1.500 | 0.267 | 2121.636 | 43.056 | 28 |
| M_1_F_qt_Set_2 | 1.485 | 0.267 | 2122.716 | 44.136 | 36 |
| M_2_F_lp_Set_1 | 1.414 | 0.267 | 2123.132 | 44.553 | 14 |
| M_3_F_qp_Set_1 | 1.465 | 0.267 | 2124.148 | 45.569 | 12 |
| M_3_F_qp_Set_2 | 1.492 | 0.200 | 2124.518 | 45.938 | 9 |
| M_1_F_lp_Set_2 | 1.468 | 0.200 | 2125.065 | 46.485 | 13 |
| M_3_F_qpth_Set_1 | 1.514 | 0.267 | 2126.052 | 47.472 | 29 |
| M_2_F_pth_Set_1 | 1.458 | 0.267 | 2126.943 | 48.363 | 33 |
| M_2_F_lqpth_Set_1 | 1.507 | 0.267 | 2127.103 | 48.523 | 33 |
| M_3_F_lp_Set_1 | 1.383 | 0.267 | 2127.974 | 49.394 | 10 |
| M_1_F_lqt_Set_2 | 1.518 | 0.267 | 2128.070 | 49.491 | 36 |
| M_0.5_F_lp_Set_2 | 1.457 | 0.200 | 2128.866 | 50.286 | 17 |
| M_2_F_th_Set_1 | 1.446 | 0.267 | 2129.084 | 50.504 | 33 |
| M_3_F_pth_Set_2 | 1.500 | 0.267 | 2129.423 | 50.843 | 28 |
| M_2_F_qpth_Set_1 | 1.511 | 0.267 | 2131.639 | 53.059 | 34 |
| M_2_F_ph_Set_2 | 1.489 | 0.267 | 2133.518 | 54.938 | 31 |
| M_2_F_lph_Set_2 | 1.501 | 0.267 | 2136.522 | 57.942 | 32 |
| M_2_F_lp_Set_2 | 1.454 | 0.267 | 2137.268 | 58.689 | 12 |
| M_3_F_lpth_Set_2 | 1.488 | 0.267 | 2137.276 | 58.696 | 30 |
| M_2_F_lth_Set_1 | 1.469 | 0.267 | 2137.373 | 58.793 | 35 |
| M_3_F_lph_Set_2 | 1.495 | 0.267 | 2138.328 | 59.748 | 29 |
| M_2_F_h_Set_2 | 1.508 | 0.267 | 2139.196 | 60.616 | 32 |
| M_2_F_lpth_Set_2 | 1.479 | 0.267 | 2140.560 | 61.980 | 34 |
| M_1_F_qt_Set_1 | 1.464 | 0.267 | 2141.148 | 62.568 | 41 |
| M_1_F_lt_Set_2 | 1.514 | 0.333 | 2142.392 | 63.813 | 39 |
| M_2_F_th_Set_2 | 1.498 | 0.267 | 2143.184 | 64.605 | 34 |
| M_2_F_lpth_Set_1 | 1.463 | 0.267 | 2144.749 | 66.169 | 36 |
| M_3_F_th_Set_2 | 1.500 | 0.267 | 2145.076 | 66.496 | 31 |
| M_3_F_lp_Set_2 | 1.433 | 0.267 | 2146.741 | 68.162 | 11 |
| M_2_F_pth_Set_2 | 1.500 | 0.267 | 2148.970 | 70.390 | 35 |
| M_3_F_lth_Set_2 | 1.480 | 0.267 | 2149.384 | 70.804 | 32 |
| M_2_F_lh_Set_1 | 1.481 | 0.267 | 2149.971 | 71.391 | 35 |
| M_1_F_lqt_Set_1 | 1.508 | 0.333 | 2153.779 | 75.199 | 42 |
| M_1_F_lqpt_Set_2 | 1.535 | 0.333 | 2155.682 | 77.102 | 40 |
| M_2_F_lh_Set_2 | 1.457 | 0.267 | 2156.286 | 77.706 | 35 |
| M_1_F_lt_Set_1 | 1.504 | 0.267 | 2160.187 | 81.608 | 43 |
| M_0.5_F_p_Set_1 | 1.371 | 0.200 | 2160.322 | 81.742 | 16 |
| M_1_F_qpt_Set_2 | 1.536 | 0.333 | 2161.312 | 82.732 | 41 |
| M_1_F_lqph_Set_1 | 1.472 | 0.200 | 2163.127 | 84.547 | 40 |
| M_1_F_pt_Set_2 | 1.523 | 0.333 | 2166.631 | 88.051 | 42 |
| M_1_F_p_Set_1 | 1.370 | 0.200 | 2169.104 | 90.524 | 15 |
| M_2_F_lth_Set_2 | 1.493 | 0.267 | 2169.297 | 90.717 | 38 |
| M_0.5_F_q_Set_1 | 1.339 | 0.200 | 2178.814 | 100.235 | 10 |
| M_1_F_pt_Set_1 | 1.453 | 0.267 | 2179.667 | 101.087 | 45 |
| M_1_F_lpt_Set_2 | 1.525 | 0.333 | 2180.375 | 101.795 | 43 |
| M_1_F_q_Set_1 | 1.347 | 0.200 | 2180.580 | 102.000 | 9 |
| M_3_F_q_Set_1 | 1.349 | 0.200 | 2181.367 | 102.787 | 6 |
| M_2_F_q_Set_1 | 1.357 | 0.200 | 2181.507 | 102.928 | 7 |
| M_1_F_lqph_Set_2 | 1.525 | 0.267 | 2183.837 | 105.257 | 41 |
| M_0.5_F_q_Set_2 | 1.352 | 0.200 | 2184.081 | 105.501 | 8 |
| M_3_F_q_Set_2 | 1.357 | 0.200 | 2184.126 | 105.546 | 4 |
| M_1_F_q_Set_2 | 1.351 | 0.200 | 2185.424 | 106.844 | 7 |
| M_2_F_q_Set_2 | 1.358 | 0.200 | 2187.247 | 108.668 | 6 |
| M_2_F_p_Set_1 | 1.336 | 0.200 | 2187.657 | 109.077 | 14 |
| M_1_F_lpt_Set_1 | 1.495 | 0.267 | 2194.748 | 116.168 | 46 |
| M_3_F_p_Set_1 | 1.296 | 0.200 | 2197.985 | 119.405 | 11 |
| M_1_F_lqh_Set_2 | 1.527 | 0.267 | 2204.426 | 125.847 | 43 |
| M_1_F_qpt_Set_1 | 1.512 | 0.267 | 2205.699 | 127.119 | 47 |
| M_1_F_lqpt_Set_1 | 1.507 | 0.267 | 2208.300 | 129.720 | 47 |
| M_0.5_F_p_Set_2 | 1.322 | 0.200 | 2212.215 | 133.636 | 15 |
| M_1_F_h_Set_2 | 1.494 | 0.267 | 2214.138 | 135.558 | 44 |
| M_2_F_qth_Set_1 | 1.469 | 0.267 | 2216.623 | 138.043 | 44 |
| M_1_F_p_Set_2 | 1.310 | 0.200 | 2226.544 | 147.964 | 13 |
| M_0.5_F_l_Set_2 | 1.388 | 0.067 | 2227.742 | 149.162 | 9 |
| M_0.5_F_l_Set_1 | 1.351 | 0.067 | 2231.753 | 153.174 | 11 |
| M_1_F_l_Set_2 | 1.410 | 0.133 | 2234.328 | 155.748 | 10 |
| M_1_F_lpth_Set_1 | 1.471 | 0.267 | 2235.236 | 156.656 | 49 |
| M_1_F_l_Set_1 | 1.335 | 0.133 | 2235.864 | 157.284 | 11 |
| M_1_F_lqh_Set_1 | 1.500 | 0.200 | 2237.659 | 159.080 | 47 |
| M_1_F_h_Set_1 | 1.468 | 0.200 | 2238.156 | 159.576 | 47 |
| M_1_F_th_Set_2 | 1.466 | 0.267 | 2244.000 | 165.420 | 48 |
| M_2_F_l_Set_2 | 1.441 | 0.133 | 2244.138 | 165.558 | 10 |
| M_1_F_lqth_Set_2 | 1.511 | 0.267 | 2244.236 | 165.656 | 48 |
| M_3_F_l_Set_2 | 1.437 | 0.067 | 2245.197 | 166.618 | 8 |
| M_2_F_l_Set_1 | 1.355 | 0.133 | 2245.915 | 167.336 | 11 |
| M_3_F_l_Set_1 | 1.385 | 0.067 | 2246.499 | 167.919 | 9 |
| M_1_F_ph_Set_2 | 1.494 | 0.267 | 2248.835 | 170.255 | 47 |
| M_1_F_lqpth_Set_1 | 1.455 | 0.267 | 2250.148 | 171.568 | 50 |
| M_1_F_lth_Set_1 | 1.464 | 0.267 | 2251.832 | 173.252 | 50 |
| M_1_F_lpth_Set_2 | 1.498 | 0.267 | 2256.111 | 177.532 | 49 |
| M_1_F_lth_Set_2 | 1.498 | 0.267 | 2256.867 | 178.288 | 49 |
| M_2_F_p_Set_2 | 1.311 | 0.200 | 2258.009 | 179.429 | 12 |
| M_1_F_lh_Set_2 | 1.480 | 0.267 | 2261.662 | 183.082 | 48 |
| M_1_F_qph_Set_2 | 1.532 | 0.267 | 2261.682 | 183.103 | 48 |
| M_1_F_qph_Set_1 | 1.485 | 0.200 | 2262.568 | 183.988 | 49 |
| M_1_F_lh_Set_1 | 1.477 | 0.200 | 2263.589 | 185.009 | 49 |
| M_3_F_p_Set_2 | 1.295 | 0.133 | 2266.651 | 188.071 | 9 |
| M_1_F_lqth_Set_1 | 1.483 | 0.267 | 2268.158 | 189.579 | 51 |
| M_1_F_lqpth_Set_2 | 1.531 | 0.267 | 2271.084 | 192.504 | 50 |
| M_1_F_lph_Set_2 | 1.503 | 0.267 | 2276.261 | 197.682 | 49 |
| M_1_F_pth_Set_1 | 1.434 | 0.267 | 2283.229 | 204.649 | 52 |
| M_1_F_qpth_Set_1 | 1.467 | 0.267 | 2301.233 | 222.653 | 53 |
| M_1_F_qpth_Set_2 | 1.510 | 0.267 | 2304.199 | 225.619 | 52 |
| M_1_F_th_Set_1 | 1.477 | 0.267 | 2323.462 | 244.882 | 54 |
| M_3_F_qph_Set_2 | 1.518 | 0.200 | 2329.570 | 250.991 | 50 |
| M_1_F_pth_Set_2 | 1.502 | 0.267 | 2367.896 | 289.316 | 55 |
| M_0.5_F_lqh_Set_2 | 1.529 | 0.267 | 2373.066 | 294.486 | 55 |
| M_1_F_lph_Set_1 | 1.467 | 0.200 | 2374.588 | 296.008 | 55 |
| M_1_F_ph_Set_1 | 1.446 | 0.200 | 2399.820 | 321.240 | 56 |
| M_1_F_qth_Set_2 | 1.482 | 0.267 | 2418.685 | 340.105 | 57 |
| M_1_F_qth_Set_1 | 1.465 | 0.267 | 2631.489 | 552.909 | 63 |
| M_0.5_F_h_Set_2 | 1.510 | 0.267 | 2656.532 | 577.952 | 63 |
| M_1_F_qh_Set_2 | 1.528 | 0.267 | 2674.651 | 596.071 | 63 |
| M_0.5_F_lqh_Set_1 | 1.502 | 0.200 | 2699.945 | 621.365 | 64 |
| M_0.5_F_lqph_Set_1 | 1.508 | 0.200 | 2764.338 | 685.758 | 65 |
| M_0.5_F_ph_Set_1 | 1.488 | 0.200 | 2843.822 | 765.243 | 66 |
| M_0.5_F_lqph_Set_2 | 1.513 | 0.267 | 2858.876 | 780.296 | 66 |
| M_1_F_qh_Set_1 | 1.470 | 0.200 | 2868.472 | 789.892 | 66 |
| M_0.5_F_lh_Set_1 | 1.495 | 0.200 | 2939.277 | 860.697 | 67 |
| M_0.5_F_h_Set_1 | 1.502 | 0.200 | 2940.321 | 861.741 | 67 |
| M_0.5_F_lph_Set_1 | 1.485 | 0.200 | 3185.934 | 1107.354 | 69 |
| M_0.5_F_lh_Set_2 | 1.517 | 0.267 | 3203.580 | 1125.000 | 69 |
| M_0.5_F_t_Set_2 | 1.530 | 0.333 | 3306.354 | 1227.774 | 70 |
| M_0.5_F_qh_Set_1 | 1.481 | 0.200 | 3360.644 | 1282.064 | 70 |
| M_0.5_F_qph_Set_2 | 1.520 | 0.267 | 3371.135 | 1292.555 | 70 |
| M_0.5_F_qh_Set_2 | 1.512 | 0.267 | 3890.151 | 1811.571 | 72 |
| M_3_F_qph_Set_1 | 1.515 | 0.200 | 3938.495 | 1859.915 | 72 |
| M_0.5_F_lph_Set_2 | 1.522 | 0.267 | 4294.704 | 2216.125 | 73 |
| M_0.5_F_ph_Set_2 | 1.513 | 0.267 | 4294.704 | 2216.125 | 73 |
| M_0.5_F_t_Set_1 | 1.538 | 0.267 | 4822.732 | 2744.152 | 74 |
| M_0.5_F_lt_Set_2 | 1.537 | 0.333 | 4844.613 | 2766.034 | 74 |
| M_0.5_F_qt_Set_2 | 1.536 | 0.333 | 4846.804 | 2768.224 | 74 |
| M_0.5_F_qph_Set_1 | 1.496 | 0.200 | 4897.875 | 2819.295 | 74 |
| M_0.5_F_lqt_Set_2 | 1.538 | 0.333 | 7927.086 | 5848.506 | 76 |
| M_0.5_F_lt_Set_1 | 1.548 | 0.400 | 14063.157 | 11984.578 | 77 |
| M_0.5_F_lqth_Set_1 | 1.569 | 0.333 | NA | NA | 82 |
| M_0.5_F_lqpt_Set_1 | 1.553 | 0.267 | NA | NA | 82 |
| M_0.5_F_lqt_Set_1 | 1.553 | 0.267 | NA | NA | 78 |
| M_0.5_F_lqpt_Set_2 | 1.549 | 0.333 | NA | NA | 83 |
| M_0.5_F_lqpth_Set_2 | 1.547 | 0.333 | NA | NA | 91 |
| M_2_F_qph_Set_2 | 1.544 | 0.267 | NA | NA | 80 |
| M_0.5_F_qpt_Set_1 | 1.539 | 0.267 | NA | NA | 82 |
| M_0.5_F_qt_Set_1 | 1.539 | 0.267 | NA | NA | 79 |
| M_0.5_F_lqth_Set_2 | 1.538 | 0.333 | NA | NA | 86 |
| M_0.5_F_lpth_Set_1 | 1.537 | 0.333 | NA | NA | 85 |
| M_0.5_F_qpt_Set_2 | 1.534 | 0.333 | NA | NA | 83 |
| M_0.5_F_lqpth_Set_1 | 1.533 | 0.267 | NA | NA | 81 |
| M_0.5_F_lth_Set_1 | 1.532 | 0.333 | NA | NA | 84 |
| M_0.5_F_qpth_Set_2 | 1.531 | 0.333 | NA | NA | 91 |
| M_0.5_F_th_Set_2 | 1.531 | 0.333 | NA | NA | 84 |
| M_0.5_F_qth_Set_2 | 1.529 | 0.333 | NA | NA | 86 |
| M_0.5_F_lth_Set_2 | 1.528 | 0.333 | NA | NA | 82 |
| M_0.5_F_qpth_Set_1 | 1.528 | 0.267 | NA | NA | 81 |
| M_0.5_F_qth_Set_1 | 1.525 | 0.267 | NA | NA | 82 |
| M_0.5_F_lpt_Set_2 | 1.520 | 0.333 | NA | NA | 78 |
| M_0.5_F_pth_Set_2 | 1.517 | 0.333 | NA | NA | 87 |
| M_0.5_F_lpt_Set_1 | 1.515 | 0.333 | NA | NA | 79 |
| M_0.5_F_pt_Set_2 | 1.513 | 0.333 | NA | NA | 78 |
| M_0.5_F_lpth_Set_2 | 1.513 | 0.333 | NA | NA | 87 |
| M_0.5_F_th_Set_1 | 1.512 | 0.333 | NA | NA | 83 |
| M_3_F_qh_Set_2 | 1.511 | 0.200 | NA | NA | 89 |
| M_2_F_qph_Set_1 | 1.511 | 0.267 | NA | NA | 87 |
| M_0.5_F_pt_Set_1 | 1.510 | 0.267 | NA | NA | 79 |
| M_2_F_qh_Set_2 | 1.507 | 0.267 | NA | NA | 78 |
| M_0.5_F_pth_Set_1 | 1.505 | 0.333 | NA | NA | 85 |
| M_2_F_qh_Set_1 | 1.493 | 0.267 | NA | NA | 78 |
| M_3_F_qh_Set_1 | 1.484 | 0.200 | NA | NA | 91 |

**Table S2 Maxent calibration results for *Globodera rostochiensis***. Totally 248 candidate models were evaluated. Candidate models were ranked by AICc.

| Model | Mean AUC ratio of pROC | Omission rate at 5% | AICc | Delta AICc | Number of parameters |
| --- | --- | --- | --- | --- | --- |
| M_2_F_t_Set_1 | 1.423 | 0.212 | 4439.497 | 0.000 | 36 |
| M_2_F_lt_Set_1 | 1.418 | 0.212 | 4439.497 | 0.000 | 36 |
| M_2_F_t_Set_2 | 1.428 | 0.152 | 4443.809 | 4.312 | 30 |
| M_2_F_lt_Set_2 | 1.424 | 0.152 | 4446.568 | 7.071 | 31 |
| M_2_F_qt_Set_1 | 1.442 | 0.212 | 4446.734 | 7.237 | 35 |
| M_3_F_t_Set_1 | 1.426 | 0.182 | 4448.615 | 9.119 | 31 |
| M_3_F_qpth_Set_1 | 1.445 | 0.182 | 4448.982 | 9.485 | 25 |
| M_2_F_pt_Set_2 | 1.410 | 0.212 | 4451.135 | 11.638 | 31 |
| M_3_F_lqth_Set_1 | 1.431 | 0.152 | 4452.040 | 12.543 | 26 |
| M_3_F_lqt_Set_1 | 1.474 | 0.212 | 4453.122 | 13.626 | 26 |
| M_2_F_qpt_Set_1 | 1.451 | 0.212 | 4453.317 | 13.821 | 37 |
| M_3_F_qt_Set_1 | 1.472 | 0.212 | 4454.071 | 14.575 | 27 |
| M_2_F_lqt_Set_1 | 1.446 | 0.212 | 4454.426 | 14.930 | 37 |
| M_3_F_lt_Set_1 | 1.432 | 0.182 | 4454.721 | 15.224 | 33 |
| M_3_F_qpt_Set_1 | 1.471 | 0.212 | 4456.854 | 17.357 | 28 |
| M_3_F_lqpth_Set_1 | 1.434 | 0.152 | 4457.531 | 18.035 | 28 |
| M_2_F_qt_Set_2 | 1.466 | 0.152 | 4457.567 | 18.071 | 28 |
| M_3_F_qth_Set_1 | 1.441 | 0.182 | 4457.883 | 18.387 | 28 |
| M_2_F_lqpt_Set_1 | 1.448 | 0.212 | 4458.075 | 18.578 | 38 |
| M_2_F_qpth_Set_1 | 1.433 | 0.182 | 4458.236 | 18.739 | 36 |
| M_3_F_lqpt_Set_1 | 1.476 | 0.212 | 4458.824 | 19.328 | 28 |
| M_3_F_qt_Set_2 | 1.489 | 0.152 | 4458.942 | 19.445 | 19 |
| M_2_F_pt_Set_1 | 1.416 | 0.212 | 4460.205 | 20.708 | 41 |
| M_2_F_lqt_Set_2 | 1.469 | 0.152 | 4460.621 | 21.124 | 29 |
| M_3_F_t_Set_2 | 1.438 | 0.152 | 4460.982 | 21.486 | 28 |
| M_3_F_lt_Set_2 | 1.436 | 0.152 | 4460.982 | 21.486 | 28 |
| M_2_F_qpt_Set_2 | 1.466 | 0.152 | 4463.102 | 23.606 | 30 |
| M_3_F_qpt_Set_2 | 1.487 | 0.152 | 4463.889 | 24.393 | 21 |
| M_2_F_qth_Set_1 | 1.437 | 0.182 | 4464.161 | 24.664 | 38 |
| M_2_F_lpt_Set_1 | 1.427 | 0.212 | 4464.739 | 25.242 | 42 |
| M_3_F_lpt_Set_1 | 1.443 | 0.182 | 4465.094 | 25.597 | 32 |
| M_2_F_lqpth_Set_1 | 1.425 | 0.182 | 4465.224 | 25.728 | 38 |
| M_3_F_lqt_Set_2 | 1.497 | 0.152 | 4465.637 | 26.141 | 21 |
| M_2_F_lqpt_Set_2 | 1.470 | 0.152 | 4466.172 | 26.675 | 31 |
| M_3_F_th_Set_1 | 1.411 | 0.212 | 4466.911 | 27.414 | 32 |
| M_3_F_lth_Set_2 | 1.487 | 0.091 | 4467.576 | 28.079 | 24 |
| M_3_F_th_Set_2 | 1.488 | 0.091 | 4467.604 | 28.107 | 24 |
| M_2_F_lpt_Set_2 | 1.390 | 0.182 | 4467.730 | 28.233 | 36 |
| M_3_F_lqpt_Set_2 | 1.493 | 0.152 | 4467.789 | 28.293 | 22 |
| M_1_F_qpt_Set_1 | 1.416 | 0.212 | 4469.414 | 29.918 | 56 |
| M_3_F_pt_Set_1 | 1.433 | 0.182 | 4469.459 | 29.963 | 33 |
| M_3_F_lqth_Set_2 | 1.504 | 0.121 | 4470.660 | 31.163 | 22 |
| M_3_F_pth_Set_1 | 1.417 | 0.212 | 4470.753 | 31.256 | 33 |
| M_0.5_F_lq_Set_1 | 1.434 | 0.121 | 4471.405 | 31.909 | 16 |
| M_2_F_lqth_Set_1 | 1.428 | 0.182 | 4471.415 | 31.919 | 40 |
| M_3_F_pt_Set_2 | 1.420 | 0.121 | 4471.427 | 31.930 | 28 |
| M_1_F_t_Set_2 | 1.432 | 0.182 | 4471.497 | 32.001 | 51 |
| M_2_F_pth_Set_1 | 1.422 | 0.182 | 4471.548 | 32.052 | 40 |
| M_2_F_lth_Set_1 | 1.412 | 0.182 | 4474.365 | 34.868 | 41 |
| M_2_F_th_Set_1 | 1.415 | 0.212 | 4474.640 | 35.144 | 41 |
| M_0.5_F_lqp_Set_1 | 1.415 | 0.121 | 4475.376 | 35.879 | 21 |
| M_3_F_pth_Set_2 | 1.475 | 0.152 | 4476.476 | 36.980 | 27 |
| M_3_F_lpth_Set_2 | 1.484 | 0.152 | 4476.488 | 36.991 | 27 |
| M_1_F_lq_Set_1 | 1.446 | 0.121 | 4476.821 | 37.324 | 15 |
| M_3_F_qph_Set_1 | 1.438 | 0.121 | 4477.072 | 37.575 | 25 |
| M_3_F_lqpth_Set_2 | 1.508 | 0.121 | 4477.911 | 38.415 | 25 |
| M_3_F_lqh_Set_1 | 1.436 | 0.152 | 4478.071 | 38.575 | 26 |
| M_1_F_lqp_Set_1 | 1.441 | 0.121 | 4478.606 | 39.110 | 19 |
| M_3_F_lpt_Set_2 | 1.423 | 0.121 | 4478.987 | 39.490 | 29 |
| M_1_F_qt_Set_1 | 1.423 | 0.212 | 4479.500 | 40.004 | 58 |
| M_3_F_qh_Set_1 | 1.443 | 0.121 | 4480.245 | 40.749 | 26 |
| M_1_F_lpt_Set_2 | 1.415 | 0.182 | 4480.695 | 41.199 | 54 |
| M_3_F_qpth_Set_2 | 1.494 | 0.152 | 4481.261 | 41.764 | 26 |
| M_2_F_lpth_Set_1 | 1.414 | 0.182 | 4482.107 | 42.611 | 43 |
| M_1_F_pt_Set_2 | 1.411 | 0.182 | 4482.117 | 42.621 | 54 |
| M_1_F_lqpt_Set_1 | 1.415 | 0.212 | 4483.116 | 43.619 | 59 |
| M_1_F_t_Set_1 | 1.398 | 0.212 | 4483.231 | 43.734 | 59 |
| M_3_F_lqph_Set_1 | 1.435 | 0.121 | 4483.814 | 44.317 | 28 |
| M_3_F_lth_Set_1 | 1.403 | 0.182 | 4485.553 | 46.057 | 38 |
| M_3_F_lpth_Set_1 | 1.408 | 0.212 | 4489.510 | 50.014 | 39 |
| M_3_F_qth_Set_2 | 1.498 | 0.121 | 4490.317 | 50.820 | 29 |
| M_3_F_lqph_Set_2 | 1.497 | 0.091 | 4491.582 | 52.085 | 23 |
| M_3_F_qph_Set_2 | 1.493 | 0.152 | 4491.582 | 52.085 | 23 |
| M_1_F_lt_Set_1 | 1.405 | 0.212 | 4492.138 | 52.642 | 61 |
| M_3_F_lqh_Set_2 | 1.496 | 0.091 | 4494.570 | 55.073 | 24 |
| M_3_F_qh_Set_2 | 1.485 | 0.152 | 4494.570 | 55.073 | 24 |
| M_0.5_F_lq_Set_2 | 1.482 | 0.121 | 4494.863 | 55.367 | 14 |
| M_0.5_F_lqp_Set_2 | 1.460 | 0.121 | 4494.956 | 55.459 | 20 |
| M_2_F_lqpth_Set_2 | 1.510 | 0.121 | 4495.556 | 56.059 | 40 |
| M_1_F_lqp_Set_2 | 1.458 | 0.091 | 4495.588 | 56.091 | 17 |
| M_3_F_h_Set_2 | 1.489 | 0.121 | 4495.618 | 56.122 | 26 |
| M_1_F_pt_Set_1 | 1.393 | 0.212 | 4496.191 | 56.694 | 62 |
| M_1_F_lq_Set_2 | 1.494 | 0.091 | 4497.327 | 57.831 | 13 |
| M_1_F_lt_Set_2 | 1.433 | 0.182 | 4497.435 | 57.939 | 57 |
| M_2_F_lq_Set_1 | 1.460 | 0.091 | 4497.721 | 58.224 | 16 |
| M_3_F_ph_Set_2 | 1.483 | 0.152 | 4497.726 | 58.230 | 27 |
| M_3_F_lh_Set_2 | 1.492 | 0.152 | 4497.913 | 58.417 | 27 |
| M_2_F_qth_Set_2 | 1.500 | 0.121 | 4497.937 | 58.441 | 41 |
| M_2_F_lph_Set_1 | 1.425 | 0.121 | 4498.148 | 58.651 | 38 |
| M_3_F_h_Set_1 | 1.435 | 0.182 | 4498.525 | 59.029 | 33 |
| M_2_F_qh_Set_2 | 1.530 | 0.152 | 4498.755 | 59.258 | 32 |
| M_1_F_lqt_Set_1 | 1.426 | 0.212 | 4498.914 | 59.417 | 62 |
| M_2_F_qpth_Set_2 | 1.505 | 0.121 | 4499.113 | 59.616 | 41 |
| M_2_F_qh_Set_1 | 1.438 | 0.121 | 4499.284 | 59.788 | 38 |
| M_2_F_pth_Set_2 | 1.479 | 0.121 | 4499.450 | 59.953 | 41 |
| M_3_F_ph_Set_1 | 1.426 | 0.182 | 4500.492 | 60.996 | 34 |
| M_3_F_lph_Set_2 | 1.479 | 0.152 | 4500.558 | 61.061 | 28 |
| M_2_F_lqh_Set_1 | 1.434 | 0.121 | 4501.517 | 62.020 | 39 |
| M_2_F_th_Set_2 | 1.478 | 0.121 | 4501.724 | 62.228 | 42 |
| M_3_F_lph_Set_1 | 1.415 | 0.152 | 4501.873 | 62.377 | 35 |
| M_2_F_lqp_Set_1 | 1.464 | 0.091 | 4502.886 | 63.390 | 20 |
| M_2_F_lqp_Set_2 | 1.478 | 0.091 | 4503.854 | 64.357 | 14 |
| M_0.5_F_lp_Set_1 | 1.361 | 0.121 | 4504.677 | 65.181 | 22 |
| M_2_F_lqph_Set_1 | 1.439 | 0.121 | 4504.699 | 65.202 | 40 |
| M_2_F_lh_Set_1 | 1.429 | 0.121 | 4505.316 | 65.819 | 40 |
| M_2_F_lth_Set_2 | 1.483 | 0.121 | 4505.412 | 65.916 | 43 |
| M_2_F_lq_Set_2 | 1.499 | 0.091 | 4505.880 | 66.384 | 12 |
| M_1_F_lpt_Set_1 | 1.410 | 0.212 | 4505.932 | 66.435 | 64 |
| M_3_F_lh_Set_1 | 1.424 | 0.182 | 4506.050 | 66.554 | 36 |
| M_1_F_lp_Set_1 | 1.365 | 0.121 | 4506.298 | 66.802 | 20 |
| M_2_F_lpth_Set_2 | 1.480 | 0.121 | 4506.374 | 66.878 | 43 |
| M_1_F_qt_Set_2 | 1.449 | 0.152 | 4508.919 | 69.422 | 59 |
| M_2_F_lqh_Set_2 | 1.535 | 0.152 | 4511.356 | 71.860 | 36 |
| M_2_F_lqth_Set_2 | 1.509 | 0.121 | 4512.352 | 72.855 | 45 |
| M_1_F_qpt_Set_2 | 1.434 | 0.182 | 4512.471 | 72.975 | 60 |
| M_1_F_lqt_Set_2 | 1.436 | 0.182 | 4512.639 | 73.143 | 60 |
| M_1_F_th_Set_1 | 1.381 | 0.212 | 4512.785 | 73.289 | 64 |
| M_2_F_ph_Set_2 | 1.529 | 0.121 | 4513.483 | 73.987 | 37 |
| M_2_F_ph_Set_1 | 1.440 | 0.121 | 4516.366 | 76.869 | 43 |
| M_1_F_lqpt_Set_2 | 1.430 | 0.182 | 4517.137 | 77.640 | 61 |
| M_3_F_lqp_Set_2 | 1.492 | 0.091 | 4518.030 | 78.533 | 14 |
| M_3_F_lq_Set_1 | 1.480 | 0.091 | 4518.499 | 79.003 | 16 |
| M_3_F_lqp_Set_1 | 1.475 | 0.091 | 4518.578 | 79.081 | 18 |
| M_2_F_h_Set_2 | 1.524 | 0.121 | 4518.593 | 79.096 | 38 |
| M_3_F_lq_Set_2 | 1.511 | 0.061 | 4518.650 | 79.154 | 12 |
| M_0.5_F_qp_Set_1 | 1.496 | 0.091 | 4519.379 | 79.883 | 23 |
| M_2_F_qph_Set_1 | 1.433 | 0.121 | 4520.540 | 81.043 | 44 |
| M_2_F_lqph_Set_2 | 1.527 | 0.152 | 4521.012 | 81.515 | 39 |
| M_2_F_qph_Set_2 | 1.525 | 0.152 | 4521.012 | 81.515 | 39 |
| M_1_F_qth_Set_1 | 1.412 | 0.212 | 4523.429 | 83.933 | 66 |
| M_2_F_lph_Set_2 | 1.527 | 0.121 | 4523.796 | 84.299 | 40 |
| M_0.5_F_lp_Set_2 | 1.380 | 0.121 | 4523.841 | 84.345 | 18 |
| M_1_F_ph_Set_1 | 1.424 | 0.182 | 4525.531 | 86.034 | 53 |
| M_2_F_h_Set_1 | 1.444 | 0.152 | 4525.682 | 86.185 | 45 |
| M_2_F_lp_Set_1 | 1.381 | 0.152 | 4526.378 | 86.881 | 21 |
| M_2_F_lh_Set_2 | 1.531 | 0.121 | 4527.474 | 87.977 | 41 |
| M_1_F_qp_Set_1 | 1.503 | 0.061 | 4529.495 | 89.998 | 21 |
| M_1_F_qh_Set_1 | 1.431 | 0.182 | 4530.413 | 90.917 | 54 |
| M_1_F_qp_Set_2 | 1.504 | 0.030 | 4530.612 | 91.115 | 17 |
| M_1_F_lqh_Set_1 | 1.420 | 0.182 | 4531.135 | 91.639 | 54 |
| M_0.5_F_qp_Set_2 | 1.508 | 0.030 | 4533.155 | 93.658 | 21 |
| M_1_F_qph_Set_1 | 1.425 | 0.182 | 4534.382 | 94.886 | 55 |
| M_1_F_lp_Set_2 | 1.373 | 0.152 | 4535.901 | 96.405 | 20 |
| M_2_F_qp_Set_1 | 1.508 | 0.061 | 4538.216 | 98.719 | 18 |
| M_1_F_lth_Set_1 | 1.394 | 0.212 | 4539.282 | 99.786 | 69 |
| M_1_F_pth_Set_1 | 1.392 | 0.212 | 4539.467 | 99.971 | 69 |
| M_2_F_qp_Set_2 | 1.505 | 0.030 | 4540.400 | 100.904 | 16 |
| M_3_F_lp_Set_1 | 1.393 | 0.121 | 4541.911 | 102.415 | 19 |
| M_2_F_lp_Set_2 | 1.368 | 0.152 | 4542.398 | 102.901 | 16 |
| M_1_F_qpth_Set_1 | 1.411 | 0.212 | 4545.687 | 106.191 | 70 |
| M_1_F_lqth_Set_1 | 1.412 | 0.212 | 4545.720 | 106.224 | 70 |
| M_1_F_lh_Set_2 | 1.491 | 0.121 | 4546.490 | 106.994 | 52 |
| M_3_F_qp_Set_1 | 1.496 | 0.061 | 4546.991 | 107.495 | 17 |
| M_3_F_qp_Set_2 | 1.503 | 0.030 | 4548.086 | 108.589 | 14 |
| M_1_F_lqph_Set_1 | 1.419 | 0.182 | 4548.743 | 109.246 | 58 |
| M_1_F_h_Set_1 | 1.427 | 0.182 | 4549.149 | 109.653 | 58 |
| M_1_F_lqpth_Set_1 | 1.409 | 0.212 | 4551.298 | 111.801 | 71 |
| M_1_F_ph_Set_2 | 1.491 | 0.121 | 4553.336 | 113.839 | 54 |
| M_1_F_lh_Set_1 | 1.421 | 0.182 | 4554.247 | 114.751 | 59 |
| M_3_F_lp_Set_2 | 1.391 | 0.152 | 4554.905 | 115.409 | 16 |
| M_1_F_qph_Set_2 | 1.491 | 0.152 | 4555.248 | 115.751 | 55 |
| M_1_F_lpth_Set_1 | 1.395 | 0.212 | 4557.235 | 117.739 | 72 |
| M_1_F_lth_Set_2 | 1.424 | 0.182 | 4558.105 | 118.608 | 68 |
| M_1_F_th_Set_2 | 1.414 | 0.182 | 4558.547 | 119.050 | 68 |
| M_1_F_h_Set_2 | 1.492 | 0.121 | 4558.630 | 119.133 | 55 |
| M_1_F_lqph_Set_2 | 1.494 | 0.121 | 4559.889 | 120.393 | 56 |
| M_1_F_lqh_Set_2 | 1.495 | 0.121 | 4561.215 | 121.719 | 56 |
| M_1_F_qth_Set_2 | 1.428 | 0.182 | 4563.641 | 124.145 | 69 |
| M_1_F_lph_Set_2 | 1.485 | 0.121 | 4567.088 | 127.592 | 57 |
| M_1_F_lqth_Set_2 | 1.429 | 0.182 | 4569.178 | 129.682 | 70 |
| M_1_F_lpth_Set_2 | 1.412 | 0.182 | 4575.309 | 135.812 | 71 |
| M_1_F_pth_Set_2 | 1.412 | 0.182 | 4575.309 | 135.812 | 71 |
| M_1_F_qh_Set_2 | 1.503 | 0.121 | 4575.426 | 135.930 | 59 |
| M_1_F_lph_Set_1 | 1.420 | 0.182 | 4579.009 | 139.512 | 64 |
| M_1_F_qpth_Set_2 | 1.435 | 0.152 | 4587.387 | 147.890 | 73 |
| M_1_F_lqpth_Set_2 | 1.427 | 0.182 | 4587.387 | 147.890 | 73 |
| M_2_F_q_Set_1 | 1.425 | 0.091 | 4596.778 | 157.282 | 11 |
| M_2_F_q_Set_2 | 1.433 | 0.030 | 4598.810 | 159.314 | 10 |
| M_0.5_F_q_Set_1 | 1.441 | 0.030 | 4599.586 | 160.089 | 14 |
| M_3_F_q_Set_1 | 1.433 | 0.091 | 4599.856 | 160.360 | 11 |
| M_1_F_q_Set_2 | 1.434 | 0.030 | 4601.459 | 161.962 | 12 |
| M_1_F_q_Set_1 | 1.428 | 0.091 | 4601.663 | 162.167 | 14 |
| M_3_F_q_Set_2 | 1.434 | 0.061 | 4601.711 | 162.214 | 10 |
| M_0.5_F_q_Set_2 | 1.438 | 0.030 | 4602.219 | 162.723 | 13 |
| M_1_F_p_Set_1 | 1.457 | 0.091 | 4611.849 | 172.352 | 18 |
| M_0.5_F_p_Set_1 | 1.448 | 0.091 | 4612.167 | 172.671 | 20 |
| M_0.5_F_ph_Set_1 | 1.373 | 0.242 | 4616.010 | 176.514 | 76 |
| M_0.5_F_lph_Set_1 | 1.366 | 0.242 | 4616.010 | 176.514 | 76 |
| M_2_F_p_Set_1 | 1.448 | 0.061 | 4623.538 | 184.041 | 17 |
| M_0.5_F_lqph_Set_2 | 1.399 | 0.212 | 4627.862 | 188.366 | 74 |
| M_0.5_F_h_Set_1 | 1.377 | 0.242 | 4630.348 | 190.851 | 78 |
| M_0.5_F_lh_Set_1 | 1.377 | 0.242 | 4630.348 | 190.851 | 78 |
| M_3_F_p_Set_1 | 1.438 | 0.061 | 4640.853 | 201.356 | 16 |
| M_0.5_F_lqh_Set_2 | 1.404 | 0.212 | 4650.208 | 210.711 | 77 |
| M_0.5_F_h_Set_2 | 1.408 | 0.212 | 4679.496 | 239.999 | 81 |
| M_0.5_F_lh_Set_2 | 1.399 | 0.212 | 4687.449 | 247.952 | 82 |
| M_0.5_F_qh_Set_1 | 1.385 | 0.242 | 4692.827 | 253.330 | 86 |
| M_0.5_F_p_Set_2 | 1.458 | 0.000 | 4694.465 | 254.968 | 18 |
| M_0.5_F_qph_Set_2 | 1.399 | 0.212 | 4701.803 | 262.307 | 84 |
| M_0.5_F_lqh_Set_1 | 1.381 | 0.242 | 4701.916 | 262.419 | 87 |
| M_0.5_F_ph_Set_2 | 1.406 | 0.182 | 4702.285 | 262.788 | 84 |
| M_1_F_p_Set_2 | 1.440 | 0.000 | 4703.718 | 264.221 | 17 |
| M_0.5_F_t_Set_2 | 1.367 | 0.242 | 4716.255 | 276.758 | 98 |
| M_2_F_p_Set_2 | 1.427 | 0.030 | 4719.346 | 279.849 | 14 |
| M_0.5_F_qph_Set_1 | 1.376 | 0.242 | 4719.664 | 280.167 | 89 |
| M_0.5_F_lqph_Set_1 | 1.376 | 0.242 | 4719.664 | 280.167 | 89 |
| M_0.5_F_lph_Set_2 | 1.393 | 0.182 | 4719.770 | 280.273 | 86 |
| M_3_F_p_Set_2 | 1.435 | 0.030 | 4725.360 | 285.864 | 12 |
| M_0.5_F_lt_Set_2 | 1.366 | 0.212 | 4727.214 | 287.717 | 99 |
| M_0.5_F_lqt_Set_2 | 1.377 | 0.212 | 4728.328 | 288.832 | 99 |
| M_0.5_F_qt_Set_2 | 1.366 | 0.212 | 4728.328 | 288.832 | 99 |
| M_0.5_F_qh_Set_2 | 1.414 | 0.212 | 4737.395 | 297.899 | 88 |
| M_0.5_F_l_Set_2 | 1.173 | 0.061 | 4737.466 | 297.969 | 13 |
| M_1_F_l_Set_2 | 1.178 | 0.061 | 4738.666 | 299.170 | 13 |
| M_0.5_F_l_Set_1 | 1.189 | 0.061 | 4738.842 | 299.345 | 14 |
| M_1_F_l_Set_1 | 1.172 | 0.061 | 4739.999 | 300.503 | 14 |
| M_2_F_l_Set_2 | 1.167 | 0.061 | 4742.960 | 303.464 | 13 |
| M_2_F_l_Set_1 | 1.158 | 0.061 | 4744.339 | 304.843 | 14 |
| M_3_F_l_Set_2 | 1.193 | 0.061 | 4747.447 | 307.951 | 12 |
| M_3_F_l_Set_1 | 1.176 | 0.061 | 4749.036 | 309.539 | 13 |
| M_0.5_F_pt_Set_2 | 1.368 | 0.182 | 4776.874 | 337.378 | 103 |
| M_0.5_F_lpt_Set_2 | 1.363 | 0.182 | 4776.874 | 337.378 | 103 |
| M_0.5_F_lqpt_Set_2 | 1.370 | 0.212 | 4790.955 | 351.459 | 104 |
| M_0.5_F_qpt_Set_2 | 1.365 | 0.212 | 4790.955 | 351.459 | 104 |
| M_0.5_F_pth_Set_2 | 1.378 | 0.273 | 4850.579 | 411.083 | 108 |
| M_0.5_F_lpth_Set_2 | 1.376 | 0.273 | 4850.579 | 411.083 | 108 |
| M_0.5_F_qpth_Set_2 | 1.375 | 0.273 | 4850.579 | 411.083 | 108 |
| M_0.5_F_lqpth_Set_2 | 1.371 | 0.273 | 4850.579 | 411.083 | 108 |
| M_0.5_F_lth_Set_2 | 1.377 | 0.273 | 4852.474 | 412.977 | 108 |
| M_0.5_F_lqth_Set_2 | 1.375 | 0.273 | 4852.474 | 412.977 | 108 |
| M_0.5_F_qth_Set_2 | 1.375 | 0.273 | 4852.474 | 412.977 | 108 |
| M_0.5_F_th_Set_2 | 1.375 | 0.273 | 4852.474 | 412.977 | 108 |
| M_0.5_F_lt_Set_1 | 1.288 | 0.303 | 5022.228 | 582.731 | 118 |
| M_0.5_F_t_Set_1 | 1.251 | 0.273 | 5022.228 | 582.731 | 118 |
| M_0.5_F_lpt_Set_1 | 1.314 | 0.303 | 5070.959 | 631.463 | 120 |
| M_0.5_F_pt_Set_1 | 1.308 | 0.303 | 5070.959 | 631.463 | 120 |
| M_0.5_F_lqt_Set_1 | 1.292 | 0.303 | 5154.638 | 715.142 | 123 |
| M_0.5_F_qt_Set_1 | 1.287 | 0.303 | 5154.638 | 715.142 | 123 |
| M_0.5_F_lqpt_Set_1 | 1.288 | 0.303 | 5184.159 | 744.663 | 124 |
| M_0.5_F_qpt_Set_1 | 1.285 | 0.303 | 5184.159 | 744.663 | 124 |
| M_0.5_F_qth_Set_1 | 1.301 | 0.303 | 5251.642 | 812.146 | 126 |
| M_0.5_F_lqth_Set_1 | 1.297 | 0.303 | 5251.642 | 812.146 | 126 |
| M_0.5_F_lth_Set_1 | 1.292 | 0.303 | 5251.642 | 812.146 | 126 |
| M_0.5_F_th_Set_1 | 1.285 | 0.303 | 5251.642 | 812.146 | 126 |
| M_0.5_F_pth_Set_1 | 1.305 | 0.303 | 5287.050 | 847.553 | 127 |
| M_0.5_F_qpth_Set_1 | 1.300 | 0.303 | 5287.050 | 847.553 | 127 |
| M_0.5_F_lqpth_Set_1 | 1.298 | 0.303 | 5287.050 | 847.553 | 127 |
| M_0.5_F_lpth_Set_1 | 1.297 | 0.303 | 5287.050 | 847.553 | 127 |
